# Vertical flow array chips reliably identify cell types from single-cell mRNA sequencing experiments

**DOI:** 10.1038/srep36014

**Published:** 2016-11-23

**Authors:** Masataka Shirai, Koji Arikawa, Kiyomi Taniguchi, Maiko Tanabe, Tomoyuki Sakai

**Affiliations:** 1 Hitachi, Ltd., Research & Development Group 1-280, Higashi-koigakubo, kokubunji-shi, Tokyo, Japan.

## Abstract

Single-cell mRNA sequencing offers an unbiased approach to dissecting cell types as functional units in multicellular tissues. However, highly reliable cell typing based on single-cell gene expression analysis remains challenging because of the lack of methods for efficient sample preparation for high-throughput sequencing and evaluating the statistical reliability of the acquired cell types. Here, we present a highly efficient nucleic reaction chip (a vertical flow array chip (VFAC)) that uses porous materials to reduce measurement noise and improve throughput without a substantial increase in reagent. We also present a probabilistic evaluation method for cell typing depending on the amount of measurement noise. Applying the VFACs to 2580 monocytes provides 1967 single-cell expressions for 47 genes, including low-expression genes such as transcription factors. The statistical method can distinguish two cell types with probabilistic quality values, with the measurement noise level being considered for the first time. This approach enables the identification of various sub-types of cells in tissues and provides a foundation for subsequent analyses.

Single-cell gene expression analysis utilizing high-throughput DNA sequencing has emerged as a powerful tool to investigate complex biological systems^1^,^2^,^3^,^4^,^5^,^6^,^7^. Such analyses provide an unbiased means of identifying various cell types in tissues to characterize multicellular biological systems^1^,^7^,^8^,^9^,^10^,^11^,^12^,^13^,^14^, as well as insight into the processes of cell differentiation^14^,^15^, genetic regulation^16^,^17^,^18^ and cellular interactions^19^,^20^,^21^ at single-cell resolution. Although cell typing without a priori knowledge provides a foundation for further studies of biological processes, including screening gene markers, the lack of statistical reliability hampers the application of single-cell analysis in discerning the functions of genes in heterogeneous tissues. To address this limitation, precise measurement technologies^11^,^20^,^22^,^23^,^24^,^25^,^26^,^27^,^28^, high-throughput sample preparation technologies^2^,^11^,^12^,^24^ and statistical methods for determining cell types^1^,^11^ have recently been developed. The measurement of gene expression in single cells intrinsically suffers from considerable measurement noise because mRNAs are present in small amounts in individual cells^22^,^23^. To alleviate the problem of noise, a sophisticated method involving unique molecular identifiers (UMIs) has been developed^25^,^26^,^27^ that effectively reduces the measurement noise caused by the PCR amplification of cDNA synthesized from mRNA. However, the measurement noise arising from the low efficiency of cDNA synthesis in a random sample of mRNAs remains significant. Another source of stochasticity in measurements is the biomolecular processes of gene expression^23^,^29^,^30^. A sufficient number of cells must be analyzed to reduce the influence of randomness. High-throughput sample preparation technologies have been employed to dissect cellular types^2^,^11^,^12^,^31^, and the simultaneous pursuit of high efficiency and high throughput in sample preparation has led to highly reliable cell typing. The resulting single-cell data are analyzed using various clustering or visualization algorithms, including hierarchical clustering^11^,^18^, principal component analysis (PCA)^4^,^12^,^18^,^32^, graph-based methods^9^,^18^,^32^, t-distributed stochastic neighbor embedding (tSNE)^1^,^7^, the visualization of high-dimensional single-cell data based on tSNE (viSNE)^33^, k-means combined with gap statistics (RaceID)^1^, and a mixed model of probabilistic distributions with information criteria or a regularization constant^11^. A statistical or probabilistic clustering method^1^,^11^ that can evaluate the reliability of clustering is desirable for comparing cell types from different experiments with different marker genes. Although various clustering indices have been reported^34^,^35^,^36^, the evaluation of clustering from different data sets remains a challenging problem, especially for noisy data^35^. In the pioneering work by Fa and Nandi^35^, these problems were addressed by introducing two tuning parameters to alleviate the problem for noisy data sets. However, this approach requires a reference data set to select the parameters, and the parameters have no geometrical meaning in the data space.

Here, to achieve high-efficiency and high-throughput sample preparation for high-throughput sequencers, we have developed a vertical flow array chip and a statistical method for evaluating the quality of clustering based on a noise model previously determined from a standard sample. The efficiency of sample preparation from standard mRNA to molecular counts with UMIs was estimated to be greater than 50 ± 16.5% for more than 15 copies of injected mRNA per microchamber. Flow-cell devices, including multiple chips, were applied to suspended cells, and 1967 cells were analyzed to discriminate between undifferentiated cells (THP1) and PMA differentiated cells. Our statistical clustering evaluation method offers the ability to determine the number of clusters without ground-truth data to supervise the evaluation; it is also based on additional information regarding measurement noise and cluster size, which controls the fractions of false elements in clusters to avoid overestimation of the number of clusters beyond the measurement resolution. It successfully provides the most probable number of clusters and is consistent with the results obtained using well-established methods, including a Gaussian mixture model with a Bayesian information criterion (BIC)^34^,^37^ and various clustering indices such as a silhouette index^36^. The method also provides quality values (pq-values) for clusters and determines different values of the most probable number of clusters depending on the degree of measurement noise and the cluster size, which controls the error rate, which is the fraction of false assignment of data to a cluster. The introduction of the two parameters controls the minimum geometrical size of clusters and the rate of false elements in clusters. Users of the statistical method can select the parameter values according to their predetermined noise model and error rate standard.

Finally, it was demonstrated that highly precise gene expression data were acquired from 76% of the dispensed cells, and two types of cells were derived with maximum pq-values among the possible number of clusters. Since it is difficult to reduce measurement noise thoroughly by improving the efficiency of sample preparation, the combination of the VFAC-based and statistical method to effectively manage the stochastic behavior of single-cell gene expression can be applied to various fields, including immunology, cancer biology, and developmental biology.

## Results

### Development of a flow-cell device for high-throughput single-cell sample preparation

**Figure 1:**
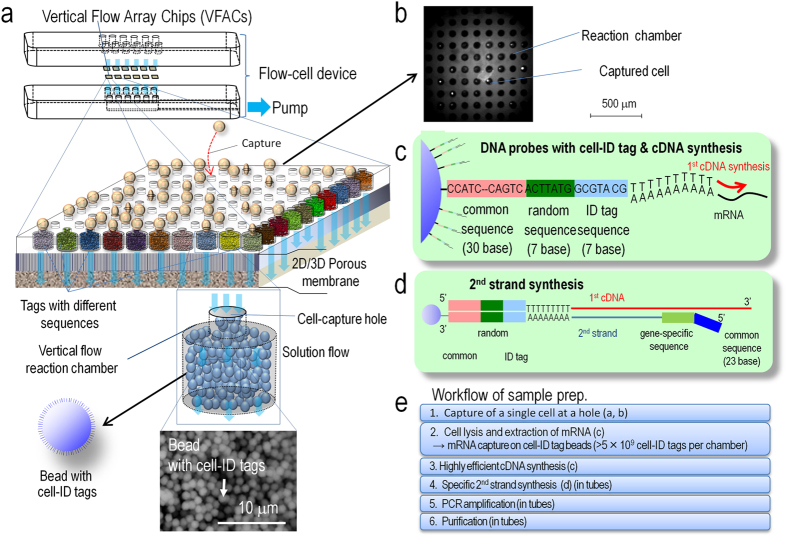
Schematic structure of the vertical flow array chip and flow-cell device. (**a**) The vertical flow chips contain 100 microchambers with beads providing porous reaction fields. Each microchamber features a different cell-id tag sequence in the DNA probes immobilized on the beads. (**b**) Fluorescence microscopy image of the VFAC with trapped cells, where small white dots indicate individual cells. (**c**) Structure of DNA probes on the beads for mRNA trapping and 1^st^ cDNA synthesis. (**d**) Schematic diagram of 2^nd^ strand synthesis on beads. (**e**) Workflow of sample preparation for gene expression analysis using VFACs. Second-strand synthesis and subsequent steps were performed in a tube containing the VFAC.

Our flow-cell device for single-cell analysis is composed of multiple VFACs to enable inexpensive, digital gene expression profiling for thousands of single cells across an arbitrary number of genes without using robotics. Figure 1 presents a schematic of the flow-cell device and the VFACs. Each chip in the device contains 100 vertical flow-through microchambers for synthesizing a cDNA library and a through-hole to capture single cells. After the single cells are drawn and captured in the holes, which are 3–5 micrometers in diameter, the drawing of lysis buffer into the holes enables the extraction of mRNA strands, which are then trapped by DNA probes with poly-T sequences immobilized on the packed beads in the microchambers. Then, 5 × 10^9^ copies of the DNA probes in each chamber (2 orders of magnitude greater than in previous works^11^) with cell-ID tags are immobilized on the 1 × 10^5^ beads composing the porous structure in each microchamber. This structure enables the highly efficient trapping of mRNA within a few minutes and highly efficient cDNA synthesis from the small amount of mRNA in single cells. The cDNA library from single cells on the beads in the VFACs is then used to construct a sequencing library for high-throughput sequencing by PCR amplification. Gene-specific probes combined with primers are used for PCR (Methods) to acquire sequencing data, and the data are sorted by the cell tag sequences to identify their original positions on the chip. Single-cell gene expression data were acquired from the cells suspended in solution buffer. The high-density microchambers on the chips and the vertical flow system reduced the volume of expensive enzymatic reagent per cell by a factor of 20^28^, thus reducing the cost of sample preparation compared to reactions performed in tubes. The microchambers on the VFACs have no walls to prevent the exchange of reagents, simplifying the structure of the VFACs and the flow-cell device, and the high-density integration of the microchambers further reduces reagent costs. The crosstalk between wells was suppressed by continuous down flow through the micro-wells.

The fabrication of the VFACs comprised injection molding of polydimethylsiloxane (PDMS) to produce microchamber arrays and laser aberration to produce through-holes to capture cells. To retain the beads in the microchambers, we used a hydrophilic porous membrane (ISOPORE membrane (Millipore)) to cover the backs of the VFACs.

Figure 1(b) presents the workflow for single-cell analysis using VFACs. To prepare the VFACs, we used an inkjet system designed for biochemical applications (Microjet Inc., LabJet 500) to dispense solutions and magnetic beads to immobilize the DNA probes. The probes consisted of 18 bp of poly-T sequence, 7 bp of cell identifier (Cell-ID) and 7 bp of random sequence as UMIs. The supply of common reagents through the micro-reaction chambers on the chip enabled cDNA synthesis with gene-specific primers after mRNA trapping. After removal of the chips from the flow-cell device, PCR reactions were performed in tubes.

### Quantification performance of the vertical flow chip

**Figure 2:**
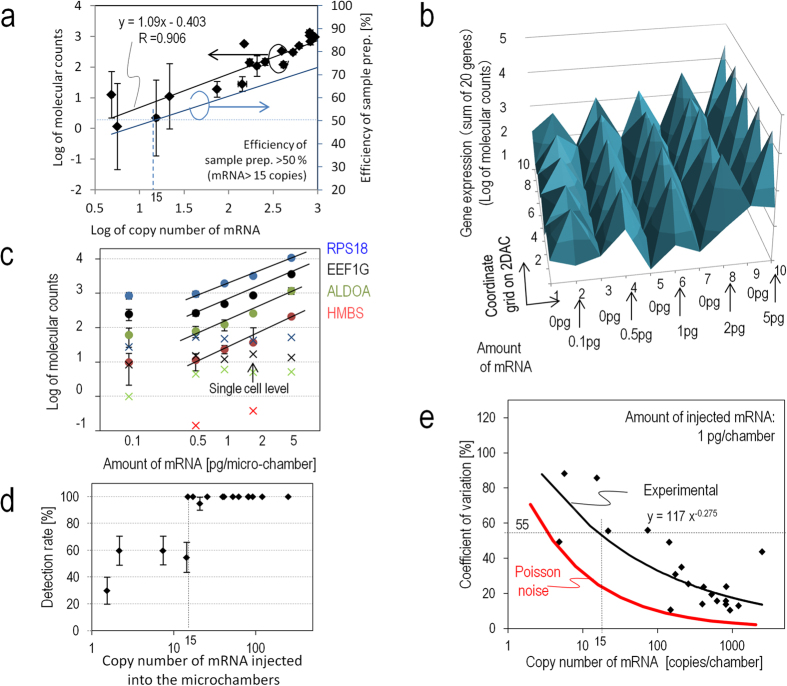
Quantitative performance of VFACs. (**a**) Reaction efficiency from mRNA to molecular counts by sequencing 20 housekeeping genes. The R value is the square root of the residual variance. (**b**) Mapping of the molecular counts on VFACs for a predetermined position and amount of injected mRNA. (**c**) Dynamic range of quantification by molecular counting of fragments covering the amount generated at the single-cell level: 1 pg of mRNA. Cross markers indicate background signals corresponding to each gene expression level indicated by the round markers. These data indicate that crosstalk during the RT reaction was less than 1%. (**d**) Minimum detection limit of this method. (**e**) Coefficient of variation of molecular counts for various levels of expression.

We tested the quantification performance of the VFACs using a calibrated amount of mRNA (Methods). The mRNA solutions were injected into microchambers on the VFACs, and the cDNAs were then synthesized individually on the chips. After PCR amplification, next-generation sequencing (NGS; Ion PGM) was performed. First, the copy numbers of mRNA determined by qPCR were correlated with molecular counts by sequencing (Fig. 2(a)). The copy numbers of mRNA injected into each microchamber on the chips were estimated using a calibration curve of the cDNA and cRNA acquired by bead-based RT-qPCR (Supplementary Figure 4). The efficiency of sample preparation from mRNA to molecular counts was estimated to be greater than 50 ± 16.5% for the injection of more than 15 copies of mRNA per microchamber (Fig. 2(a)). The acquired efficiency was approximately 4 times higher than was obtained by Drop-seq^7^,^14^.Second, we assessed the parallel sample preparation capability of the microchambers when the mRNA solutions were injected. Figure 2(b) presents the mapping of the total expression of 20 housekeeping genes on a VFAC with 10 by 10 micro-reaction chambers. The positions of the peaks matched the positions of the microchambers into which the solutions were injected, confirming the accuracy of the sample position assignments. The quantities of gene expression in Fig. 2(b–e) were indicated as molecular counts using UMIs, where the total number of acquired reads mapped on 20 genes was approximately 1 million for each VFAC. Third, Fig. 2(c) presents calibration curves of the gene expression levels of four housekeeping genes (RPS18, EEF1G, ALDOA, and HMBS). The amount of mRNA in a single cell (1 pg) is within the linear region for all genes. Figure 2(d) presents the lowest copy number detection limit of 15 copies per chamber, at which the detection rate is greater than 90%. Finally, we estimated the coefficient of variation (CV) depending on the copy number of mRNAs injected into individual chambers (Fig. 2(e)). These curves describing the CV values of measurement noise (55% at 15 copies of mRNA) are comparable to the ones reported previously^26^ as a result of the highly efficient cDNA synthesis. The regression curve was used as a probabilistic model of measurement noise.

### Description of the statistical method for calculating pq-values

The main purpose of the statistical method is the unbiased comparison of the qualities of the clusters across different experimental conditions, including measurement noise, number of cells and number of genes. For the unbiased comparison of clustering results among the different conditions, the function used for clustering evaluation should have a definite range for all conditions. Therefore, a probabilistic function is preferable for the clustering evaluation because the range of probability is from 0 to 1. Furthermore, the application of a conventional clustering evaluation method to highly stochastic single-cell gene expression data may assign high scores to accidentally formed clusters even though the cluster size is smaller than the measurement noise or have a potentially large fraction of false assignment of individual cells to clusters due to overlapping distributions. Therefore, we propose an evaluation function for clustering with a clustering resolution that considers measurement noise and a cluster size that controls the fraction of false assignment of cells to a cluster.

Our method is based on a probabilistic distribution model of gene expression and a noise model of measurement; the model assumes a multidimensional log-normal distribution^38^. An explicit parameterization of clustering resolution and clustering size is the first key step in evaluating the probability that a certain datum is appropriately allocated to a cluster. The first parameter to determine the resolution of clustering is defined as a surface with constant likelihood from a covariance matrix, which is determined from the noise model of a measurement for a standard sample. The noise model consists of measurement noise, regarded as a minimum resolution for clustering, and intrinsic biological noise, derived from the covariance matrices of acquired individual clusters. Similarly, the second parameter varies from 0 to 1 and indicates the size of a cluster with probabilistic confidence, as the pq-value of a cluster is defined as the maximum boundary with constant likelihood to control the fraction of false assignment of data to each cluster. The second key element of the method is the evaluation of both the probability that each datum belongs to a cluster (homogeneity) and the probability that the datum does not belong to other clusters (separation). These criteria permit the method to appropriately calculate the pq-values of clusters such that the method can avoid overfitting without any information criteria, as will be discussed later. An example of overfitting is that an optimum number of clusters is determined to be a number of fitting parameters in a Gaussian mixture model.

**Figure 3:**
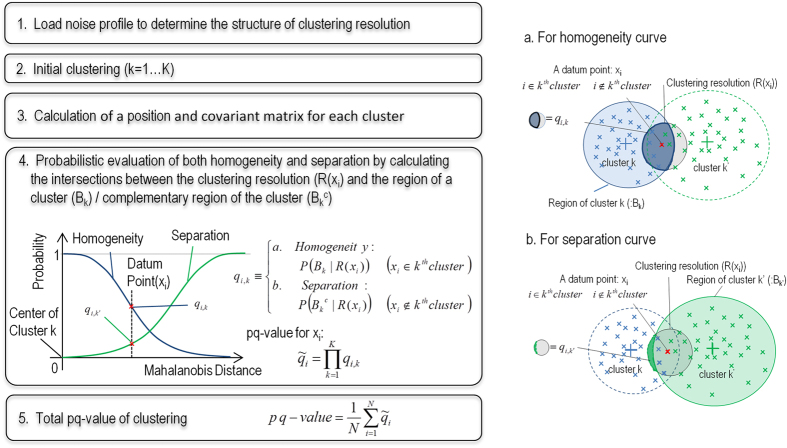
Schematic diagram of the algorithm of the probabilistic clustering method. (**a**) Block diagram of the method. (**b**) Illustration of calculations for homogeneity and separation. After evaluating the initial clustering, the centers and covariance matrices of the cluster data are replicated K-fold, where K is the number of clusters. The clustering resolutions were used to evaluate the probability of the correctness of the clustering of the data with respect to homogeneity and separation.

A schematic diagram of the algorithm for evaluating the pq-value of clustering is shown in Fig. 3. The first step is the loading of the measurement noise model, which is expressed as a fitting curve of the standard deviations against the gene expression levels for standard samples. For a given gene expression datum, the constant-confidence surface in n-dimensional space (n: number of genes) is defined as the clustering resolution of the measurement noise. Next, the acquired data are applied to a k-means or hierarchical clustering algorithm with a given number of clusters: K (1…K_max_). The center of each cluster (mean) and the eigenvalue of the covariance matrices are calculated to determine the cluster size (CS) and clustering resolution (Σ_CR_), which is determined from the measurement noise and intrinsic biological noise. The clustering resolution is defined as the largest of the variations between the resolution of biological intrinsic noise and measurement noise in each direction of the eigenvector (axis of principal components) and for each cluster (Methods).

Generally, clusters can be characterized in terms of homogeneity and separation based on the definition of the cluster^39^. For a fixed datum point x_i_ and cluster k (k = 1…K), the probability of homogeneity for cluster k (blue curve in Fig. 3) can be defined as a conditional probability of q_k_ = P(B_k_|R(x_i_)), where B_k_ is the inside region of cluster k determined by a constant Mahalanobis distance, and R(x_i_) is also an inside region determined by the clustering resolution. Similarly, the probability of separation for cluster k’, except for k (green curve in Fig. 3), can be defined as q_k’_ = P(B_k’_^c^|R(x_i_)), where B_k’_^c^ is the complementary region of B_k’_. The pq-value for a datum x_i_ is evaluated as Πq_k_ (k = 1…K), and the pq-value of clustering is evaluated based on the average of Πq_k_ over all x_i_ (Methods).

### Performance of the statistical method

**Figure 4:**
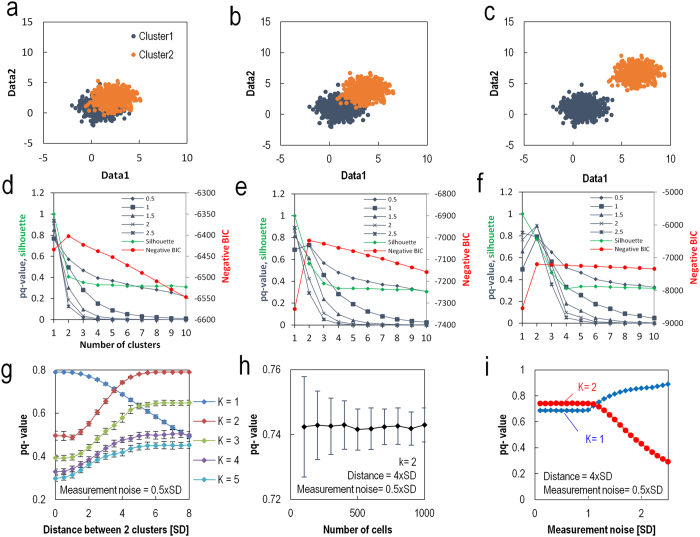
Validation of the statistical method for generated data. (**a–c**) Distribution of the generated data with various distances between the clusters; the distances are 2, 4, and 8 times the standard deviation of the cluster. There are 1000 total data points. CS = 0.9, Σ_ms_ = 0.5 SD, and dimensions = 2. The definition of the position of the cluster is shown in Supplemental Figure 1. (**d–f**) negative BIC, silhouette and pq-values for the three generated data sets against predetermined numbers of clusters. The pq-value results for a different set of parameters are shown in Supplementary Figure 3. (**g**) The pq-values for various cluster distances. The maximum pq-value occurs for K = 2 when the distance between the clusters is larger than 3 SD. (**h**) The standard deviation of the pq-values decreases as the number of cells increases. (**i**) An increase in measurement noise reduces the resolution of the clustering and reduces the optimum number of clusters from K = 2 to K = 1. The red curve represents K = 2, and the blue curve represents K = 1.

We assessed the performance of our statistical method for data generated using a mixture of Gaussian distributions with various parameters, including the number of genes (n = 2, 10, 20, 50), the number of data points (N = 100–1000), and the distance between clusters (0–8 times the standard deviation (SD) of a cluster), by comparing the optimum number of clusters with the results of other conventional methods, including the Gaussian mixture model with the negative signed BIC^34^,^37^,^40^ (which is (−1) × BIC and represented as negative BIC) and silhouette index^36^ (Fig. 4, Supplemental Figures 1 and 2). The parameters for calculating the pq-values included the measurement noise (Σ_me_ = 0.5, 1.0, 1.5, 2.0, 2.5 × SD), cluster size (CS = 90%) and number of clusters (K = 1–5). As shown in Fig. 4, the numbers of clusters for maximum pq-values when the measurement noise is less than 1.0 × SD were consistent with the numbers for negative BICs in various numbers of genes and of data points. For larger measurement noise, a single cluster was selected in the pq-value method as expected. The apparent discrepancy in the optimum number of clusters between negative BICs and pq-values when the distance between two clusters is small is related to the rate of improperly assigned cell data to a cluster, which can be evaluated as the fraction of overlapping cluster distributions (Supplementary Fig. 3). Because pq-value method false assignment rate is controlled by the parameter CS, the discrepancies should come from differences in the standard of discrimination of neighboring clusters between the BIC and pq-value. The robustness of pq-values against skewing of the data distribution from the Gaussian distribution model also makes it different from the BIC (Supplementary Figure 4), as the pq-value does not rely on fitting the data to a model distribution.

In Fig. 4(g), the dependence of the pq-values for various K numbers on the distance between the clusters indicates a crossover of the maximum pq-value curves corresponding to a change in the optimum number of clusters from 1 to 2 at a distance of 3.5 SD, which depends on CS (Supplementary Figure 3). Furthermore, as the number of data points in a cluster increases, the standard deviation of the pq-values decreases (Fig. 4(h)). As measurement noise increases, the pq-value of clustering decreases when the measurement noise is larger than the SD (Fig. 4(i)). This dependence indicates that reducing the measurement noise associated with sample preparation and increasing the cell number using a high-throughput method would be effective in improving the probabilistic quality of clustering.

### Application of the VFACs and statistical methods to cultured cells

**Figure 5:**
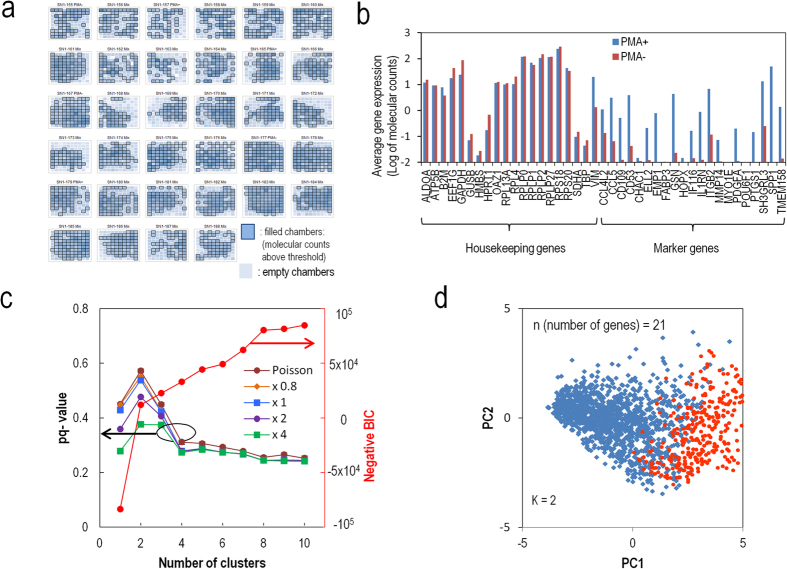
Application of the method to the cell typing of cultured cells. (**a**) Map of the microchambers in which molecular counts above a certain threshold were acquired. Here, 76% of cells in the dispensed solutions were processed successfully. (**b**) Average of molecular counts for the gene expression profiles from the PMA-positive and -negative cells. The 21 marker genes differed in their expression levels. (**c**) A negative BIC and pq-values for 1967 single cells under various cluster number settings; pq-values for various measurement noises are also shown (CS = 0.9). A clear peak in the pq-values can be observed for 2 clusters when the measurement noise level is less than 2 times the experimental noise level. (**d**) Visualization of the 2 clusters as determined by pq-values in two dimensions by principal component analysis. Blue and red dots indicate undifferentiated and differentiated cells, respectively.

Multiple (10–12) chips embedded in the flow-cell devices were used for single-cell analysis of THP1 (PMA(−)) and PMA-induced THP1 (Macrophage-like PMA(+)) cells. We injected 2580 fluorescently labeled cells into 34 VFACs (Supplementary Table 1). Because the full volume of the cellular suspension (2 μl) dispensed onto each chip was completely absorbed through the cell-capturing holes within 2 minutes, a high proportion (76%) of the dispensed cells was captured effectively (Fig. 5(a)) and generated molecular counts for gene expression greater than the threshold of 250 total molecular counts for housekeeping genes. In a previous study, the efficiency of cell capture was approximately 10–15%^7^,^12^,^14^, and the threshold for further analyses was specified as 30 total molecular counts^12^. As a result, 3.5 M molecular counts (19 M reads) were mapped onto 41 genes, including 20 housekeeping genes and 21 marker genes. The 21 genes used as markers of differentiation for THP1 were selected based on the results of a protocol for genome-wide single-cell analysis using a cDNA library on beads (bead-seq)^41^. The averaged profiles of PMA(+/−) cells demonstrated that the expression levels of 21 marker genes differed consistently with the results of the genome-wide analysis (Fig. 5(b), Methods). Successfully acquired single-cell gene expression data included low-expression genes and transcription factors (POU6F1) as low as several copies per cell, whose gene expression levels were evaluated by the average of the transcriptomes at the single-cell level (bead-seq^41^ for 34-cells (THP1), Supplementary Figure 15).

Before clustering analyses, the acquired gene expression data were pre-processed to reduce the influence of background signals (signal-to-background ratio: 8.7 ± 1.2; Supplementary Figure 5). The clustering evaluation results are presented as pq-values and the negative BICs for the 1967 cells and the 21 marker genes against the number of clusters (k). The curves of the pq-values exhibit a clear peak at k = 2 (pq-value = 53.8%), which indicates the most probable number of clusters, whereas the negative BIC curves exhibit no clear peak (Fig. 5(c)). We also evaluated the pq-values when the amounts of measurement noise were varied by multiplying the experimental noise by different factors. Although it was impossible to determine the positions of the microchambers into which PMA-induced cells were absorbed when the solution dispensed on the chip was a mixture of both types of cells, it was possible to determine whether the cell was PMA-positive or -negative by VFAC ID (6 of the 34 chips received PMA-positive or -negative cells, while the other 28 received cell mixtures; Supplementary Table 1). Because the cells from the PMA-positive and -negative chips were divided purely by clustering, we were able to identify the clusters composed of differentiated cells (Supplementary Figure 7). Figure 5(d) presents a visualization of the obtained clusters in two-dimensional space based on PCA, where red dots indicate differentiated cells. Based on cell typing, the differentiation efficiency was estimated to be 58.7%.

Increased measurement noise reduces the pq-values, indicating a negative impact of the low precision of gene expression on cell typing. Therefore, the reduction of measurement noise by highly efficient sample preparation was effective for cell typing with high reliability.

Finally, although the pq-value for the 21 genes exhibited a peak at two clusters, the selection of the marker genes to reduce the number of markers according to the expression ratio of PMA(+) to PMA(−) destabilized and changed the position of the peak of the pq-values to k = 3 (Supplementary Figure 8). This change in the position of the peak based on the selection of marker genes may indicate expansion of the clustering capability at higher resolution. Because the pq-values enable quantitative comparison between the results of clustering for different numbers of genes, the analysis of pq-values for a large number of cells with VFACs can be effective for the selection of markers, depending on the overall purpose of an analysis.

## Discussion

We have demonstrated a method for reliable cell typing based on single-cell gene expression analysis. Our approach constitutes a highly efficient and high-throughput sample preparation method on VFACs and a statistical method for evaluating the pq-values of clusters to compare clustering from different data sets, especially with different degrees of measurement noise.

The flow-cell device with the VFACs employs a batch reaction system that reduces the amount of reagents required for sample preparation for single-cell analysis. The system satisfies both the high-density alignment of the reaction chamber for reduced running costs and for highly efficient data acquisition by flowing the solution perpendicular to the chip surface through the porous structure. This vertical flow system enables the removal of potential inhibitors for 1^st^-strand synthesis in cell suspensions. Furthermore, the multiple flows through the microchambers reduce the time required to isolate cells and extract mRNA to below 10 minutes, thus decreasing the potential for variation in gene expression profiles during cell handling.

The number of cells used in a single flow-cell device easily permits scaling to up to as many as 100 chips (10,000 cells) through the use of chip identification tags during PCR amplification. Furthermore, gene expression analysis of all genes can be performed on the VFAC platform by modifying the sample preparation process to introduce poly-A tails on the 5′ ends of the cDNAs.

Our statistical method, using the parameters of clustering resolution and cluster size, identifies the optimum number of clusters controlling the rate of false classification in the clusters. These parameters characterize the pq-values from conventional methods such as BIC and other clustering evaluation methods. This study is the first to quantitatively link the probabilistic quality of clustering to the measurement noise level arising from sample preparation. It is also important to control the false assignment rate because clustering in single-cell gene expression analyses should be performed as an initial analysis and following analyses, such as a comparison of gene expression levels among different clusters, in which homogeneity in clusters is assumed. This method represents a powerful approach to excluding low-quality data and identifying sets of genes by characterizing cluster discrimination based on pq-values.

The combination of our statistical method and VFACs will be applied to identify unbiased cell types with a reliability that can be cross-validated between experiments with different conditions. It will also be applied to identify multiple gene markers that characterize both unknown and well-known cell types with high confidence.

## Methods

### Preparation of VFACs

To prepare the VFAC, a 1-mm square chip with an array of microchambers was fabricated by the injection molding (Fluidware Technologies, Inc. (Kasukabe-shi, Saitama-ken, Japan) of polydimethylpolysiloxane (PDMS). In the array, 75-μm diameter microchambers with a depth of 70 μm were aligned in square grids at 125-μm intervals. Laser ablation processing (L. P. S. Works Co. Ltd. (Ohta-ku, Tokyo, Japan)) was performed to produce through-holes with diameters of 5–7 μm in the bottoms of the microchambers for cell capture. DNA probes with various types of cell tags were immobilized on magnetic beads (Thermo Fisher Scientific, MyOne Streptavidin C1). An inkjet printer designed for biological applications (Microjet Corp. LabJet 500) was used to inject 9.6 nl of bead solution containing 1% glycerol, 1–2 × 10^5^ beads and 10 mM Tris HCl (pH 7.5) into each microchamber. The solvent of the injected solution was absorbed by a hydrophilic aluminum oxide porous membrane filter (GE Healthcare, Anopore (0.2-μm diameter pores)) at the bottom of the microchambers. The printer injected separately prepared bead solutions with different cell ID tags into predetermined microchambers such that the VFAC contained 100 microchambers with 100 cell-ID tags. Each microchamber also featured a porous surface with 5–10 × 10^9^ high-density mRNA trapping probes per chamber.

### Assembling the flow-cell device

The flow-cell device consisted of upper and lower plates fabricated from polymethylmethacrylate (PMMA) (FUKOKU BUSSAN Co., Ltd. (Ohta-ku, Tokyo, Japan)), with the VFACs between the two plates. As shown in the schematic structure of the flow-cell device in Fig. 1, the upper plate contained chambers for each VFAC to hold various solutions, including the cellular solution and RT reagents. The lower plate was used to apply negative pressure to the rear side of the VFAC to aspirate the various solutions through the VFACs and was aligned with vertical holes positioned on the VFACs and the corresponding chambers. We inserted a porous membrane (EMD Millipore, Isopore membrane ATTP 04700) to retain the DNA-immobilized beads in the microchambers between the VFACs and the bottom plate. We also used silicon rubber rings with a thickness of 0.05 mm to apply pressure and prevent leakage between the VFACs and the plates. Additionally, we embedded another type of porous membrane (SPG technology Co. Ltd., SPG membrane, 2 and 10 μm pore diameter, 0.7 mm thickness, hydrophilic surface) to equalize the negative pressure in the VFAC to capture individual cells. Finally, the 2 plates were clamped using three screws to ensure tight contact between the pieces.

### Cell culture and induced macrophage differentiation

To induce macrophage differentiation, approximately 2 × 10^6^ THP1 cells were seeded in 25 cm^2^ flasks (SUMILON) and cultured in 5 ml of RPMI 1640 supplemented with 10% fetal bovine serum (FBS) containing 160 nM phorbol-12-myristate-13-acetate (PMA) under 5% CO_2_ at 37 °C for 48 hours. As a control, 2 × 10^6^ THP1 cells were seeded in 25-cm^2^ flasks and cultured in 5 ml of RPMI 1640 supplemented with 10% FBS under 5% CO_2_ at 37 °C for 48 hours. After rinsing once with PBS, the PMA-treated THP1 macrophages were incubated at 37 °C for 5 minutes with 0.75 ml of Accumax (Funakoshi). Then, 2 ml of medium was added to deactivate the enzymes, and the cell suspension was centrifuged at 700 rpm for 3 minutes at 4 °C in a 50-ml tube. The control THP1 cell suspension was also centrifuged at 700 rpm for 3 minutes at 4 °C in a separate tube. The supernatants were removed from both tubes, and the cell pellets were resuspended in serum-free RPMI 1640.

Then, 5 μl of Vybrant DiO cell-labeling solution (Thermo Fisher) was added to both cell suspensions containing 10^6^ cells and mixed thoroughly. After incubation for 20 minutes at 37 °C, the suspensions were centrifuged at 700 rpm for 2 minutes at 4 °C. The supernatants were removed from both tubes, and the cells were washed with 1 ml of PBS. The washing processes were repeated two more times. After removal of the supernatants, the cells were diluted with PBS to produce cell suspensions (70–100 cells/μl).

### Capturing cells and cDNA synthesis on VFACs

A schematic diagram of the procedures from cell capture to PCR amplification is shown in Supplementary Figure 9. The steps from cell capture to cDNA synthesis were performed in the flow-cell device. The remaining steps were performed after placing the VFAC in a tube. Prior to cell capture, 1.0 μl of PBS buffer was added to each VFAC assembled in the flow-cell device. The device was prepared for cell capture by adding 1.0 μl of cell suspension (70–100 THP1 cells) with PBS to each VFAC. The cells were labeled with a fluorescent agent (Thermo Fisher Scientific, Vybrant DiO cell-labeling solution, excitation: 494 nm, emission: 520 nm). The flow-cell device was connected to a diaphragm pump (Ulback KOKI, Inc., DMA-015) to apply negative pressure for 2 minutes to the rear side of the VFACs, to immobilize the cells in the cell-capture holes. The cell-capture process was observed using a fluorescence microscope (Olympus, BX51N-34-FLD-1) and CCD camera (Andor Technology, iXon + DU-888E). We added 2.0 μl of PBS buffer to each VFAC and allowed the PBS buffer to adsorb for 3 minutes to remove residual cells from the chips. We also added 0.5 μl of cell-lysis solution (mixture of 0.475 μl of lysis buffer (Roche, Real Time Ready Cell Lysis Kit) and 0.025 μl of RNase inhibitor (Roche)) to the VFACs and continued pumping for 5 minutes at room temperature.

After cell lysis, 5.0 μl of reverse transcription reagent (a mixture of 0.68 μl of D.W. (Ambion), 1.0 μl of 5x FS buffer, 1.0 μl of 10 mM dNTPs, 0.66 μl of RNase OUT (Thermo Fisher) and 0.66 μl of Super Script III (Thermo Fisher)) was added to each VFAC in the flow-cell device. After sealing the inlets of the flow-cell device with adhesive film (Optical Adhesive Film, Thermo Fisher Scientific), the device was incubated for 5 minutes at 37 °C followed by 50 minutes at 50 °C in a thermostatic incubator (Titec, M.BR-022UP). The VFACs were then removed from the device and placed on a cover glass (0.12–0.17 mm thickness, Matsunami glass). We then placed silicon rubber rings (4 mm inner diameter, 1 mm thickness) around the individual VFACs and another cover glass on the rings to cover the VFACs. The rings and cover glasses were treated with RNase ZAP (Thermo Fisher Scientific) and washed with sterilized water before use. After deactivating the enzymes for reverse transcription by heating the glass for 90 seconds at 85 °C (Applied Biosystems, Gene Amp System 9700), we added 1.0 μl of reagent (a mixture of 0.5 μl of RNase buffer and 0.5 μl of RNase H) to the individual VFACs. The VFACs were then incubated for 30 minutes at 37 °C in a thermostatic incubator.

We prepared 50 μl of 10 mM Tris buffer (pH 8.0) with 0.1% Tween 20 in 200 μl tubes and then placed the VFACs in individual tubes. An NdFeB magnet (Hitachi Metals) was placed on the side wall of the tube to remove the cDNA-immobilized beads from the VFACs, and the beads were resuspended. The supernatant was removed while the beads were immobilized on the side of the tube by the magnet. After spinning down the chip and the beads, the remaining supernatant was removed again. Then, we added 1.0 μl of solution buffer (0.1% Tween 20 and 10 mM Tris-HCl (pH 8.0)) and resuspended the beads.

### Second cDNA synthesis

We dispensed 10.0 μl of the second cDNA synthesis reagent (a mixture of 2.5 μl of D.W., 1.0 μl of 10 × HF PCR buffer, 1.0 μl of 2.5 mM dNTPs, 0.4 μl of 50 mM MgSO_4_, 5 μl of a 47-plex primer mixture (each 2.5 μM) and 0.1 μl of Platinum Taq DNA polymerase High Fidelity (Thermo Fisher)) on ice and then resuspended the beads. Next, second-strand synthesis was performed using cycles of 10 seconds at 98 °C, 60 seconds at 43 °C, and 3 minutes at 68 °C, followed by incubation at 10 °C in a thermal cycler (ABI 9700, ramp rate: 9600 mode). The supernatant was removed while the beads were kept on the surface of the tube by the magnet, on ice. The cDNA-immobilized beads were then washed twice with 50 μl of washing buffer (0.1% Tween 20 and 10 mM Tris-HCl (pH 8.0)). After the washing buffer was removed, the cDNA-immobilized beads were dispersed in 1.0 μl of resuspension buffer (10 mM Tris-HCl (pH 8.0)).

### PCR amplification

We added PCR reaction reagent (mixture of 7.0 μl of 2 × Gflex buffer, 2 μl of the 5′ side of the PA primer (10 μM), 2.0 μl of the 5′ side of the P1 primer (10 μM), 0.3 μl of Gflex DNA polymerase (Takara Bio) and 3.0 μl of distilled water). PCR amplification was performed using the following thermal cycling parameters: 98 °C for 10 seconds, followed by 3 cycles of 98 °C for 10 seconds, 61 °C for 15 seconds, and 68 °C for 15 seconds; 3 cycles of 98 °C for 10 seconds, 59 °C for 15 seconds, and 68 °C for 15 seconds; 3 cycles of 98 °C for 10 seconds, 57 °C for 15 seconds, and 68 °C for 15 seconds; and 15 cycles of 98 °C for 10 seconds, 55 °C for 15 seconds, and 68 °C for 15 seconds, followed by 68 °C for 3 minutes and incubation at 4 °C. We used touchdown PCR conditions to increase specificity. After PCR amplification, 15 μl of the supernatant containing the target amplicons was transferred to a 1.5-ml tube using the magnet. After adding 35 μl of washing buffer (0.1% Tween 20 and 10 mM Tris-HCl (pH 8.0)) to the original PCR tube, we washed the beads and tube surface and again added the supernatant to the 1.5-ml tube (50 μl total).

### Purification of PCR products

Residual enzymes, primers and amplified short fragments were removed using a PCR Purification kit (Qiagen), and the purified PCR product was eluted in 50 μl of washing buffer (0.1% Tween 20 and 10 mM Tris-HCl (pH 8.0)). We also used 55% guanidinium chloride after DNA immobilization to improve the efficiency of purification. We used 60 μl of Ampure bead solution (Beckman Coulter) to remove short fragments of DNA with lengths below 100 bp.

### Preparation of diluted mRNA

Total RNA (30 μg) was extracted from 2 × 10^6^ THP1 cells using an RNeasy mini kit (QIAGEN). After DNase treatment, the mRNA was purified using an Oligotex-dT30 kit (TaKaRa Bio), and phenol/chloroform extraction was performed again. After ethanol precipitation, the concentrations of mRNA were estimated based on UV absorption. The extracted mRNA was diluted to 0.5 pg/μl, and 1 μl of this solution was used to compare RT-qPCR with our method.

### Preparation of RT probe-immobilized beads

Streptavidin-coated beads (2 × 10^9^ beads; ϕ = 1 μm, Dynal, MyOne C1) were suspended in 200 μl of binding buffer after being washed three times with 200 μl of buffer. A solution containing an RT probe (5′-modification: dual biotin; spacer: C6; Integrated DNA Technologies) was diluted with binding buffer to obtain 200 μl of RT probe solution (5 × 10^11^ molecules/μl). The RT probe solution was then added to the same volume of streptavidin-coated beads and mixed at 750 rpm at 37 °C for 1 hour. After being washed three times with 200 μl of binding buffer, the beads were transferred to a new tube (because some amount of the RT probe adsorbs on the inner wall of the tube). Then, the beads were washed three more times with 200 μl of washing buffer. After the buffer was removed, the beads were suspended in 200 μl of washing buffer. We confirmed that nearly all input RT probes were immobilized on the beads by qPCR measurement of the unreacted probes in the washing buffer.

### RT-qPCR of diluted mRNA in tubes for copy-number references

All processes for producing the cDNA library (RT) were performed in one tube to minimize sample loss. First, we dispensed 1.5 μl of RT probe-immobilized beads (10^7^ beads/μl, 5 × 10^4^ RT probes immobilized per bead); then, 1 μl of mRNA (100 pg/μl, equivalent to a single-cell) was added to the tube and mixed with the beads. We added 5.0 μl of reverse transcription reagent (mixture of 0.68 μl of distilled water (Ambion), 1.0 μl of 5 × FS buffer, 1.0 μl of 10 mM dNTPs, 0.66 μl of RNase OUT (Thermo Fisher) and 0.66 μl of Super Script III (Thermo Fisher)), mixed well, and incubated the tube for 5 minutes at 37 °C and for 50 minutes at 50 °C in a thermostatic incubator (Titec, M.BR-022UP). After cDNA synthesis, the tube was heated for 90 seconds at 85 °C (Applied Biosystems, Gene Amp System 9700) to deactivate the enzymes. The cDNA-immobilized beads were then washed with 50 μl of washing buffer (0.1% Tween 20 and 10 mM Tris-HCl (pH 8.0)). After the washing buffer was removed, the cDNA-immobilized beads were dispersed in 2 μl of washing buffer. The expression levels of the 20 housekeeping genes were analyzed using a qPCR system (Applied Biosystems ABI PRISM 7500). The qPCR analysis was performed with a 20-μl solution containing 1 × Premix Ex *Taq*, (Takara-bio), 1 μM of each primer pair, and 0.25 μM MGB fluorogenic probe. The standard DNA templates and a cDNA library were analyzed simultaneously by measuring the fluorescence during thermal cycling (95 °C for 20 seconds, followed by 40 cycles of 95 °C for 5 seconds and 60 °C for 30 seconds) to obtain amplification plots. The number of target molecules in the cDNA library was estimated from the standard curves. The sequences of the PCR primers and MGB fluorescence probes and the sizes of the products are presented in Supplementary Table 4.

### RT-qPCR of mRNA on VFACs

We injected 40-nl droplets of dilute mRNA (2 ng/ml) into the microchambers on the VFACs. Because a single droplet contains 0.1 pg of mRNA, we varied the number of droplets injected into the microchambers to control the amount of mRNA injected, ranging from 0.1 to 5 pg. The cDNA synthesis and subsequent procedures were performed as previously described.

### Preparation of standard DNA templates for qPCR

Twenty types of biotinylated PCR products were produced using the primers listed in Table 6. Streptavidin-coated beads (5 × 10^8^ beads, Dynal, MyOne C1) were suspended in 50 μl of binding buffer (20 mM Tris-HCl (pH 8.0), 0.5 mM EDTA, 1 M NaCl) after washing with 50 μl of this buffer three times. The biotinylated PCR products were diluted with binding buffer and mixed to obtain a solution containing 10^6^/μl of each of the product molecules. The PCR products were immobilized on the beads by adding 50 μl of the PCR solution to the same volume of streptavidin-coated beads and then mixing them at 750 rpm in a microincubator (Titec, M.BR-022UP) at 37 °C for 1 hour. The immobilization efficiency (approximately 95%) was estimated using qPCR to measure the amounts of DNA in the solution before and after immobilization. The DNA-immobilized beads were washed three times with 100 μl of binding buffer. After removal of the buffer, the beads were also washed three times with 100 μl of washing buffer (0.1% Tween 20 and 10 mM Tris-HCl (pH 8.0)). The amount of each type of dsDNA immobilized on the beads was estimated to be 9.5 × 10^5^ molecules per 10^7^ beads. A ten-fold dilution series (a sequence of mixtures of DNA-immobilized beads and intact beads) was produced by repeatedly diluting the sample with washed intact beads. We prepared bead solutions containing the same number of beads (10^7^ beads) with different amounts of the standard DNA templates (immobilized on the beads) at concentrations ranging from 9.5 molecules to 9.5 × 10^5^ molecules per 10^7^ beads.

### Preparation of model cRNA samples for evaluating RT efficiency

The PCR products (*φX174*) were amplified with a forward primer anchored to a T7 promoter sequence and a reverse primer anchored to an oligo(dT)_30_ sequence. The sequences of the PCR primers and the sizes of the products are listed in Supplementary Table 6.

After examining the products with a bioanalyzer (Agilent), the excess primers were removed from the products using the QIAquick PCR Purification Kit. After ethanol precipitation, the DNA concentrations were measured by UV absorption.

RNA was synthesized by incubating 500 ng of PCR product at 37 °C for 1 hour in a 10-μl reaction mixture containing 90 nmol of dATP, dCTP, dGTP, and dUTP; 10 nmol of DTT; 1 μl of AmpliScribe T7-*Flash* Enzyme (EPICENTRE Biotechnologies); and 1 × AmpliScribe buffer. The RNA samples were purified by DNase and protease K treatments, followed by two extractions with phenol/chloroform according to the standard procedure. The residual dNTP in the purified RNA samples was then removed with an Oligotex-dT30 kit (TaKaRa Bio), and phenol/chloroform extraction was performed again. After ethanol precipitation, the pellets were resuspended in 100 μl of RT-PCR grade water (Life Technologies), and the RNA concentrations were measured by UV absorption. The subsequent procedures were identical to the ones previously described except that the qPCR primers described in Supplementary Table 7 were used.

### Selection of 21 marker genes by bead-Seq

We prepared 5 cultured cells with different induction times of 0, 0.5, 2.0, 24, and 72 h by PMA from THP1 according to the previously described methods. We acquired whole-genome gene expression data at the single-cell level for 34 samples using the bead-Seq method^41^. Then, we evaluated the following differentiation marker indices for every gene, defined by the slopes of the regression lines from the data of fragments per kilobase of transcript per million fragments mapped (FPKMs) versus the induction time divided by the averages of the standard deviations of FPKMs at each induction time, and sorted the data in descending order of the indices. Supplementary Table 9 presents the indices for the selected 21 marker genes.

### DNA sequencing

An Ion PGM system (Thermo Fisher Scientific) was used to sequence the PCR amplicons. Before sequencing, the PCR products were quantified by chip electrophoresis (Agilent Technology, Inc., Bioanalyzer 2100) to dilute the sample to an appropriate concentration of target fragments (26 pM). Emulsion PCR (Thermo Fisher Scientific, The Ion One Touch system 2) was performed separately for the samples from the VFACs to prepare them for sequencing. A 318 chip and 200-bp sequencing reagents were used for sequencing. The sample types (PMA(−): without induction, PMA(+): with induction, mixture of PMA(+) and PMA(−)) of the VFACs and the numbers of cells dispensed on the chips are listed in Supplementary Table 1.

### Data analysis

The fastq files of the sequencing results were acquired using the accessory software of the Ion PGM system with recalibration set to “off” and qualification filtering disabled. We created a program to categorize the acquired reads according to the tag sequences (cell-IDs and UMIs) and aligned them onto the sequences of the transcripts. The program categorizes all of the reads into various types (Supplementary Table 10) and extracts the read counts and molecular counts (number of UMIs) for each gene, cell and VFAC, as shown in Supplementary Figure 10.

The microchambers on the VFACs were discriminated by the molecular counts of the chambers in which the total molecular counts of the housekeeping genes were above a threshold determined from a histogram of the counts (Supplementary Figure 11). The remaining empty microchambers were used to estimate the background expression levels of the marker genes by averaging the levels over the empty chambers. The background profile, shown in Supplementary Figure 12, was subtracted from the individual gene expression level for each gene. The average signal-to-background ratio for molecular counts of more than 15 counts/gene/microchamber was 8.7 ± 1.2 (Supplementary Figure 6). Normalization was also performed for the cell-captured microchambers to ensure that the total expression counts were constant for each microchamber.

Supplementary Figure 10 presents the proportions of the types of reads and molecular counts, according to the definitions described in Supplementary Table 6. The proportion of targeted genes in all molecular counts was no less than 20%, which is quite low. Supplementary Figure 13 presents the proportion of read-types for the same sequencing library using a different sequencing platform. The proportion of the targeted reads obtained with MiSeq (Illumina Inc.) was approximately 80%, 2.5-fold greater than the value obtained using Ion PGM. Therefore, the quality of the PCR products was sufficient to obtain a large proportion of targeted reads.

The molecular counts for the housekeeping genes were correlated to the copy numbers of the cDNA and PCR products by bead-based qPCR to evaluate the distortion of the gene expression profiles of the molecular counts by this method (Fig. 2(a), Supplementary Figure 14).

### Evaluation of sample preparation efficiency

To evaluate the copy numbers of the housekeeping genes in the mRNA injected into the microchambers of the VFAC, we evaluated the copy numbers of the cDNAs of the housekeeping genes from a diluted mRNA sample and the RT efficiencies from the previously described cRNA sample using the highly efficient bead-based RT-qPCR method^28^. We evaluated the copy numbers of the cDNAs for 20 housekeeping genes in 100 pg of pooled mRNA using RT probe-immobilized beads (10^7^ beads/μl, 5 × 10^4^ RT probes immobilized per bead) in a tube. By varying the ratio of cRNA to cDNA with the same amount of RT probes, we were able to determine the copy numbers of 20 house-keeping genes in the 100 pg diluted mRNA sample. In Supplementary Figure 5, the cRNA data (blue diamond) are plotted against the amount of input RNA to determine the experimental RT efficiencies from cRNA. The regression line was calculated from these data. The mRNA data in Supplementary Figure 5 indicate the estimated RT efficiencies for the mRNAs of the 20 housekeeping genes. The average estimated RT efficiency of the mRNA was 97.0 ± 16.7%. The regression line was used to estimate the copy number in a few pg of mRNA injected into the microchambers.

We also directly evaluated the RT efficiency from cRNA in the reaction on VFACs as previously described. We injected 40 nl (2 × 10^4^ copies) of each cRNA into 50 microchambers using the inkjet instrument. Before injection, the head of the inkjet used to dispense the cRNA droplets was washed with 70 μl of cRNA solution (same concentration as the injected solution, 5 × 10^5^ copies/μl). The average RT efficiency was greater than 90% for one-sided 84% confidence (1 SD C.I.) (Supplementary Table 11).

### Algorithm for the statistical method

Our clustering evaluation method evaluates the probabilistic validity of the division of data into clusters under the assumption of a multivariate Gaussian distribution for each cluster. The acquired data are represented as an n-column vector in Euclid space, where n is the number of genes. The definition of the pq-value for datum x_i_ belonging to the k^th^ cluster is


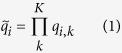


where


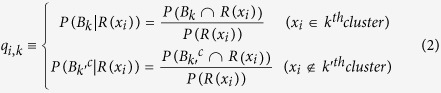



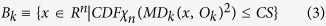


Here, 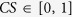 is the parameter of cluster size; 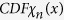 is the cumulative chi-square distribution function with n degrees of freedom; 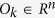 is the center (mean) of the k^th^ cluster; and 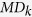 is the Mahalanobis distance determined by the covariance matrix of the k^th^ cluster. The parameter 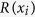 is the region of clustering resolution at 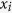, which is defined as the following representation of 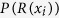:


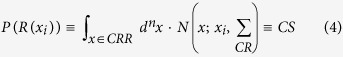



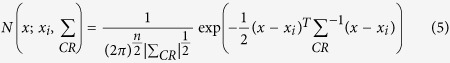


In this formula, CS also determines the region of integration involving the clustering resolution around x_i_ (CRR). The matrix 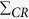 is the covariance matrix corresponding to the clustering resolution, which is defined later; 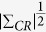 is the determinant of the covariance matrix; and (x − x_i_)^T^ is the transposed vector of (x − x_i_). In this paper, CS was set to 0.9 (90%) except when we evaluated the dependence of the pq-values on CS. Therefore, 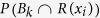 is evaluated as follows:


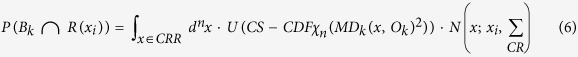



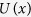
 is the unit step function, which is defined as follows:


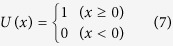


The clustering resolution in one dimension should be the sum of the square of measurement noise and the square of intrinsic noise. Essentially, we cannot know the value of intrinsic noise because we do not know the correct results of clustering. From the above description of the noises, the clustering resolution should be larger than the experimental noise. Therefore, we can construct the clustering resolution (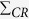) as follows. If the measurement noise (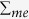) is larger than the variation of the k^th^ cluster, that is, the variance of the expressions belonging to the k^th^ cluster (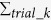), then the clustering resolution (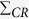) should be equal to the measurement noise (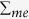). If 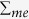 is not larger than 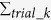, then 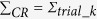. In multiple dimensions, the covariance matrix of the clustering resolution (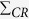) is constructed similarly, by comparing the eigenvalues of the covariance matrix of the measurement noise (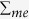) with the corresponding eigenvalues of the covariance matrix of the total noise, that is, the covariance matrix of the k^th^ trial cluster (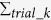), according to the following procedure:

1. Calculation of the eigenvalues and eigenvectors of 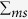 and 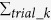.
2. Sorting the eigenvalues of the two matrices in descending order.
3. After comparison of each element of the list of eigenvalues of the two matrices, if the i^th^ element of the list from 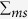 is larger than the one from 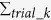, then the i^th^ element of the list from 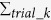 is replaced by the one from 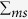.
4. Generation of a diagonal matrix with diagonal elements using the list of eigenvalues in 3.
5. Calculation of the matrix of 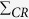 by reverse transformation of the diagonal matrix in 4 by the block matrix of eigenvectors of 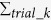.

In this paper, the model of measurement noise is determined by fitting the curve of standard deviation against the gene expression level. Therefore, the diagonal elements are equal to the square of the standard deviations. To treat the generated data, the measurement noise model is set to be constant in all directions.

To evaluate the integral in formula 6, we used the Monte Carlo algorithm, which generates virtual random data distributed on multiple Gaussian distributions with the covariance matrix 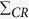.

In addition, in the evaluation of formula 6, the overlap integral 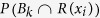 decreases with the number of genes for geometrical reasons even if the clustering, clustering resolution, and Mahalanobis distance between the center of the cluster and the data point x_i_ are fixed. Supplementary Figure 15 shows the overlap integral of 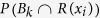 against the dimensions of space with various cluster sizes and constant distance. To compensate for this geometrical bias, we replace the value of the cluster size with the effective cluster size to retain the overlap integral as a standard value in standard dimensions (default settings: standard dimension = 1 and clustering size = 0.9 (90%)).

The implementation of the method, which is available in the Supplementary files, was performed using R.

### Validation of the statistical method

We selected the k-means algorithm for the initial clustering in LICORS (R package). The implementation of the negative BIC^34^,^37^ for the validation also used MCLUST^40^ for R. The definition of the negative BIC is following:


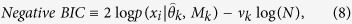


where x_i_ is observed data points, 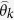 is maximizing parameter for a model M_k_, ν_k_ is the number of estimated parameter in the model M_k_ and N is the number of data points.

The other indices, including the silhouette index and the Calinski-Harabasz index, were evaluated using clusterCrit^36^ for R. Supplementary Figure 2 presents the pq-value behavior as a function of the number of cells (data) and number of dimensions (genes). Several features in the behavior of the pq-values were expected. The peaks of the pq-values against the number of clusters are very stable for changing dimensions and numbers of cells (Supplementary Figure 2). Meanwhile, as shown in Supplementary Figure 2, the negative BIC can provide 2 clusters as the optimum number of clusters even when the pq-value cannot discriminate among clusters because of their short intervals. To clarify the situation in which the negative BIC shows better performance than the pq-values, Supplementary Figure 3 shows the behavior of the optimum number of clusters in the negative BIC and pq-values against the distances between two clusters in one dimension as the simplest case. The figure also shows the fraction of overlap of the two distributions, which indicates the rate of error in assignments of the data-elements as a result of the clustering. The errors mean that a cluster includes a false data point element at the stated rate. When the value of CS is 0.1 in the pq-value, the pq-value can discriminate between two clusters at shorter distance than the negative BIC. Thus, the discrimination performance depends on the error rate. Meanwhile, a low error rate in the clusters is very important for single cell analysis because sets of data discriminated by clustering are used for further analysis, for example, gene marker analyses. In other words, simple discrimination of (the centers of) clusters is insufficient for clustering in single-cell analysis. In the pq-values, the rates of error were controlled by the value of the CS ( = 0.9).

The 3^rd^ order of skewed Gaussian data was generated in the R package (fGARCH), where a skewness of 1.0 indicates no skewness. Supplemental Figure 4 compares the robustness against the skewness between the method and the BIC. Although the pq-values were quite stable against the skewness, the number of clusters at maximum of the negative BIC varied for different skew values.

### Evaluation of cell types for cultured cells

We selected the hierarchical clustering algorithm for the initial clustering of hclust (R function). We evaluated the pq-values for various measurement noise models, among which the log-Poisson distribution was calculated using the continuous extension of the Poisson distribution by replacing the factorial with a gamma function.

Supplementary Figure 8 shows the pq-value curves as a function of the number of clusters determined by selecting genes using the descending list in Supplementary Table 9. The optimal number of clusters was destabilized when the number of genes was less than 15. When the numbers of genes were 9, 11 and 13, the optimal number of clusters became 3. Our statistical method enables comparison among the optimal numbers of clusters for different numbers of genes.

Supplementary Figure 16 presents a heat map of the 21 marker expression levels for 1967 cells. Supplementary Figure 17 presents a visualization of the clusters for 10 and 15 marker genes based on PCA.

## Additional Information

**How to cite this article**: Shirai, M. *et al.* Vertical flow array chips reliably identify cell types from single-cell mRNA sequencing experiments. *Sci. Rep.* **6**, 36014; doi: 10.1038/srep36014 (2016).

**Publisher’s note**: Springer Nature remains neutral with regard to jurisdictional claims in published maps and institutional affiliations.

## Supplementary Material

Supplementary Video

Supplementary Information

Supplementary Data
