# Supplementary material for: Vertical flow array chips reliably identify cell types from single-cell mRNA sequencing experiments
Source: Sci Rep. 2016 Nov 23;6:36014. doi: 10.1038/srep36014 (PMC5120284; doi:10.1038/srep36014)
Supplement: Supplementary Information [file srep36014-s2.pdf]

Supplementary Information

Vertical flow array chips reliably identify cell types from single-cell mRNA sequencing experiments

Masataka Shirai, Koji Arikawa, Kiyomi Taniguchi, Maiko Tanabe and Tomoyuki Sakai
[1] Hitachi, Ltd., Research & Development Group

Supplementary Note 1 | User guide for R source code and data to calculate pq-values
Supplementary Note 2 | Information on the Supplementary Video
Supplementary Figure 1 | Definition of the distance between generated clusters.
Supplementary Figure 2-1 | pq-values for various parameters.
Supplementary Figure 2-2 | Negative BICs for various distances and dimensions, with N = 1000.
Supplementary Figure 2-3 | Negative BICs for various distances and dimensions, with N = 500.
Supplementary Figure 2-4 | Negative BICs for various distances and dimensions, with N = 100.
Supplementary Figure 2-5 | Silhouette index and Calinski-Harabasz indices for various distances, with N = 1000.
Supplementary Figure 3 | Comparison of optimum number of clusters between pq-value and negative BIC for one-dimensional data
Supplementary Figure 4 | pq-values and negative BICs for generated data with various skewness levels.
Supplementary Figure 5| RT efficiency for calibrated mRNA by bead RT-qPCR of cRNA.
Supplementary Table 1. | List of VFACs and numbers of cells
Supplementary Figure 6| Signal-to-background (SB) ratio.
Supplementary Figure 7 | Dendrogram for 1967 cells with VFACs.
Supplementary Figure 8 | The pq-values for various numbers of genes.
Supplementary Figure 9 | Schematic diagram of the processes.
Supplementary Table 2 | Primer sequences for second cDNA synthesis.
Supplementary Table 3 | PCR primer sequences.
Supplementary Table 4 | RT probe sequences.
Supplementary Table 5 | Primer and probe sequences used in qPCR.
Supplementary Table 6 | PCR primer sequences for ϕX174.
Supplementary Table 7 | The qPCR primer sequences and MGB probes for ϕX174.
Supplementary Table 8 | Primer sequences for standard DNA preparations.
Supplementary Table 9| Results of bead-seq for 21 markers for PMA induction.
Supplementary Table 10 | Types of reads and their definitions.
Supplementary Figure 10 | Proportions of types of reads and derived molecular counts.
Supplementary Figure 11| Histograms of molecular counts and read counts.
Supplementary Figure 12 | Background profiles for subtraction, as determined by averaging over the microchambers below the threshold.
Supplementary Figure 13 | Comparison of the proportions of the types of reads for two types of sequencing platforms.
Supplementary Figure 14 | Correlations between molecular counts and cDNAs or PCR products for housekeeping genes in pooled mRNA samples.
Supplementary Table 11 | Direct evaluation of RT efficiency from cRNA on VFACs.
Supplementary Figure 15 | Overlap integrals for various dimensions and cluster sizes (CS).
Supplementary Figure 16 | Heat map of 21 marker expression levels in 1967 cells.
Supplementary Figure 17 | Visualization of clusters for 10 and 15 marker genes based on PCA.

Supplementary Note 1 | User guide for R source code and data to calculate pq-values

How to calculate pq-values for user data

After the preparation of matrix data (data for n genes and N cells are represented by an N-row and n-column matrix), clustering of the data should be performed by an algorithm. The clustering result is assumed to be represented by an N-column vector of which the elements are integers (1…K (K: number of clusters)) indicating clusters. The procedure to calculate the pq-values for the data and the clustering follows.

1. Copy the folder expanded from the downloaded supplementary software into your working directory, and create an output directory named "result".

2. Start R and change to your working directory.
   Input: *setwd(your_working_directory)*

3. Load pq-value functions.
   Input: *source("pqvalue.R")*

4. Calculate pq-values
   Input: *pqvalue(matrix, cluster)*
   matrix: the data matrix (N×n matrix)
   cluster: the N-column vector of the cluster index (index is an integer from 1 to K)

<u>An example of the use of the functions for user data in R</u>

```
# change directory
setwd("mydirectory")

# load library
source("pqvalue.R")

# import data matrix
data <- read.table("data.txt")

# correct clustering size
dim <- ncol(data)
cs <- approx.cs(dim)

# clustering
hcls <- hclust(dist(data), method = "ward")
cluster <- cutree(hcls, 3)

# calc pqvalue
res <- pqvalue(data, cluster, cs = cs)
pq.mean <- mean(res$pqvalue)
```

How to reproduce the pq-values in the study

All files necessary to calculate the pq-values, including the data, are contained in the Supplementary software. The procedure for reproducing pq-values in the study follows.

1. Copy the folder expanded from the downloaded supplementary software into your working directory, and create an output directory named "result".

2. Install the following R packages:
   LICORS, doSNOW, fGarch, foreach, ggplot2, GMD, gplots, mclust, reshape2

3. Start R and change to your working directory.
   Input: *setwd(your_working_directory)*

4. Load functions for analyses.
   Input: source("analysisxx.xx.R")

Supplementary List 1: List of scripts for analyses

No.	Name of script	Figures in which results are shown
1	analysis01.vdata.R	Figure 2 (a) (b) (c) (d) (e) (f)
2	analysis02.cells.R	Figure 2 (h)
3	analysis03.te.R	Figure 2 (i)
4	analysis04.skew.R	Supplementary Figure 4 (a) (b) (c)
5	analysis05.dim_dist.R	Supplementary Figure 2-1, 2-2, 2-3
6	analysis06.dim_dist.bic.R	Supplementary Figure 2-4, 2-5, 2-6
7	analysis07.dist.R	Figure 2 (g)
8	analysis11.totalnorm.R	Figure 5 (c)
9	analysis14.te.R	Figure 5 (c)
10	analysis15.ds_cells_genes.R	Supplementary Figure 8
11	analysis16.pca_scatter.R	Figure 5 (d), Supplementary Figure 17
12	analysis17.heatmap.R	Supplementary Figure 16

<u>An example of the use of the package to reproduce the results in the manuscript in R</u>

```
# change directory
setwd("mydirectory")


# load library
source("analysis01.vdata.R ")
```

Supplementary Note 2 | Information on the Supplementary Video

The video is a stream of fluorescent microscopy images whose sampling rate is 5 images per second. The format of the video is AVI, and the replay speed is 3 times faster than the acquisition speed.

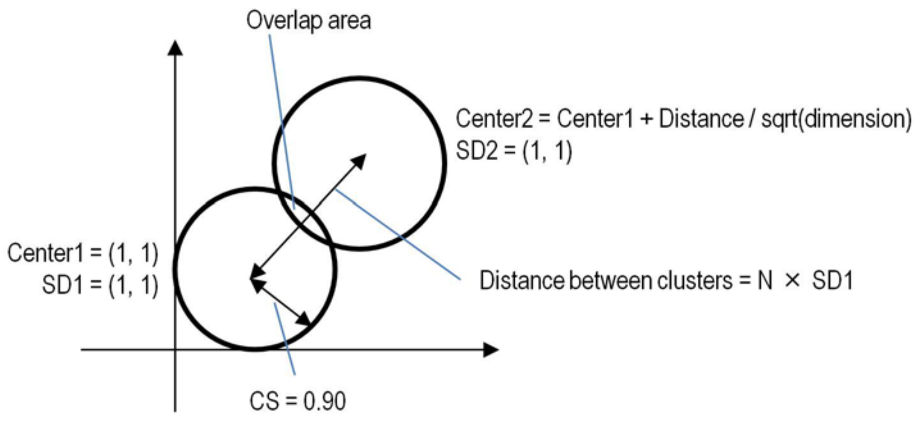

Supplementary Figure 1 | Definition of the distance between generated clusters.
Two clusters were generated in n dimensions, where Center2 = N*SD1/sqrt(n) = (1,1…,1) + (N, N…N) / sqrt(n) (n-vector).

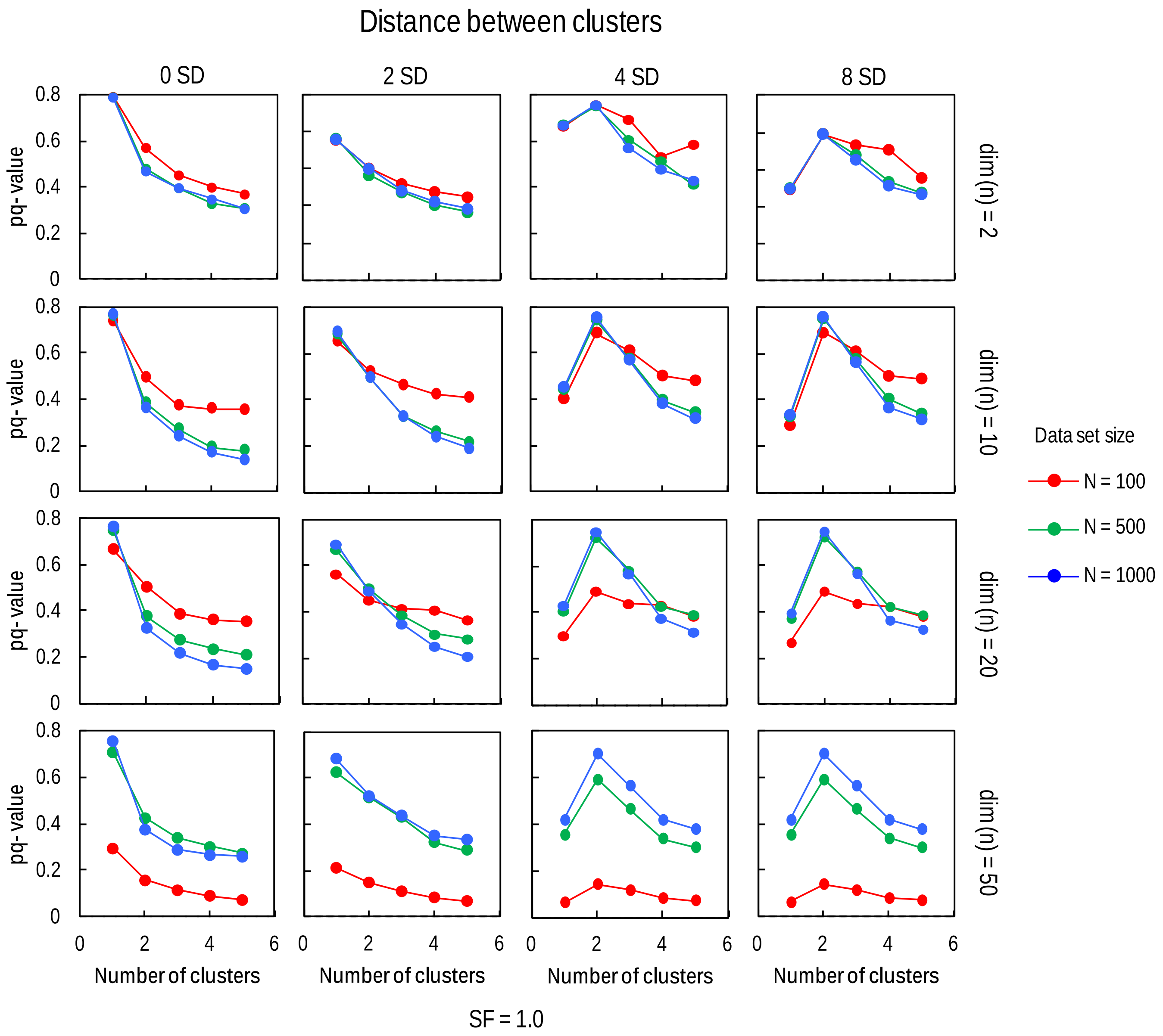

Supplementary Figure 2-1 | pq-values for various parameters.

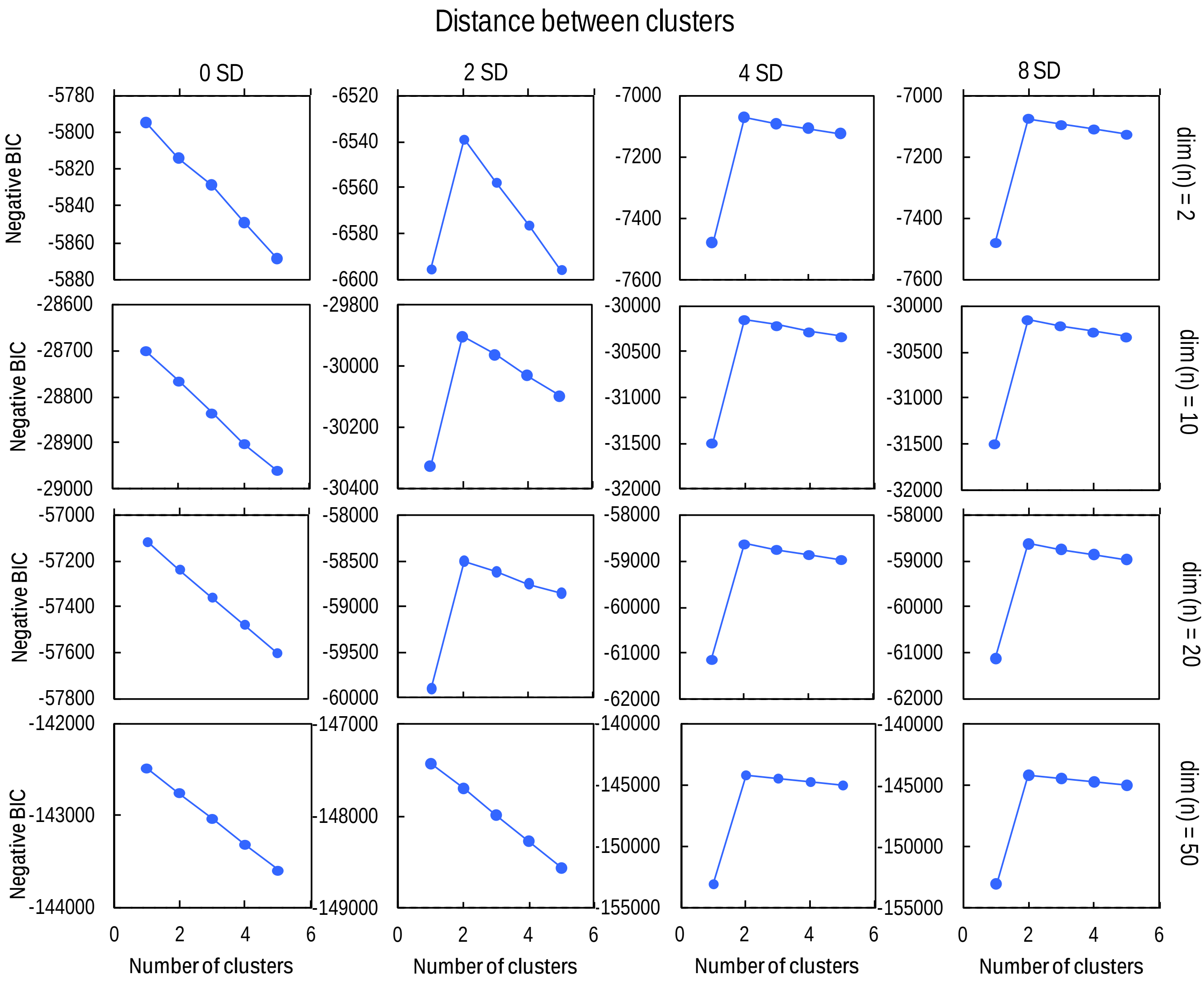

Supplementary Figure 2-2 | Negative BICs for various distances and dimensions, with N = 1000.

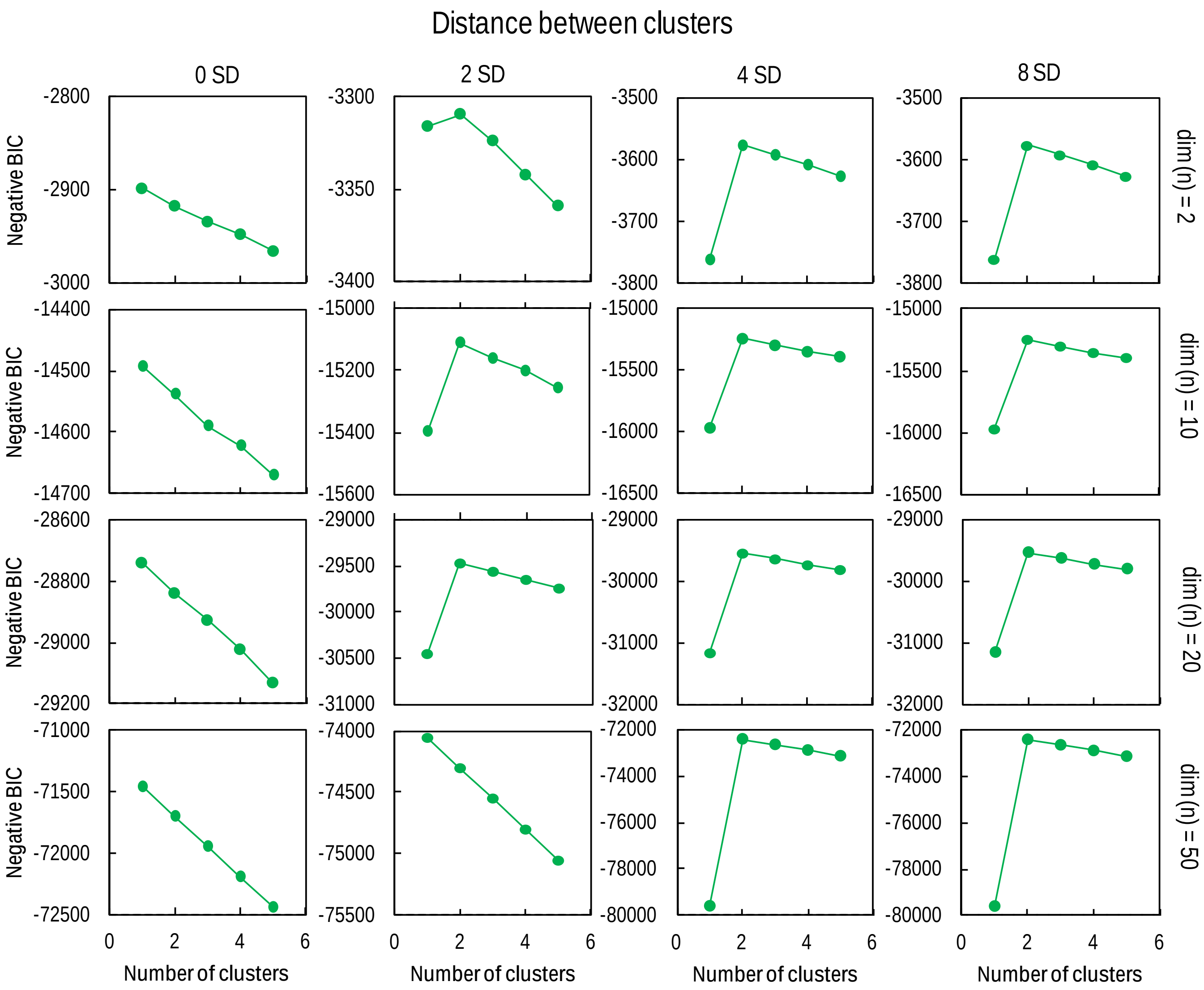

Supplementary Figure 2-3 | Negative BICs for various distances and dimensions, with N = 500.

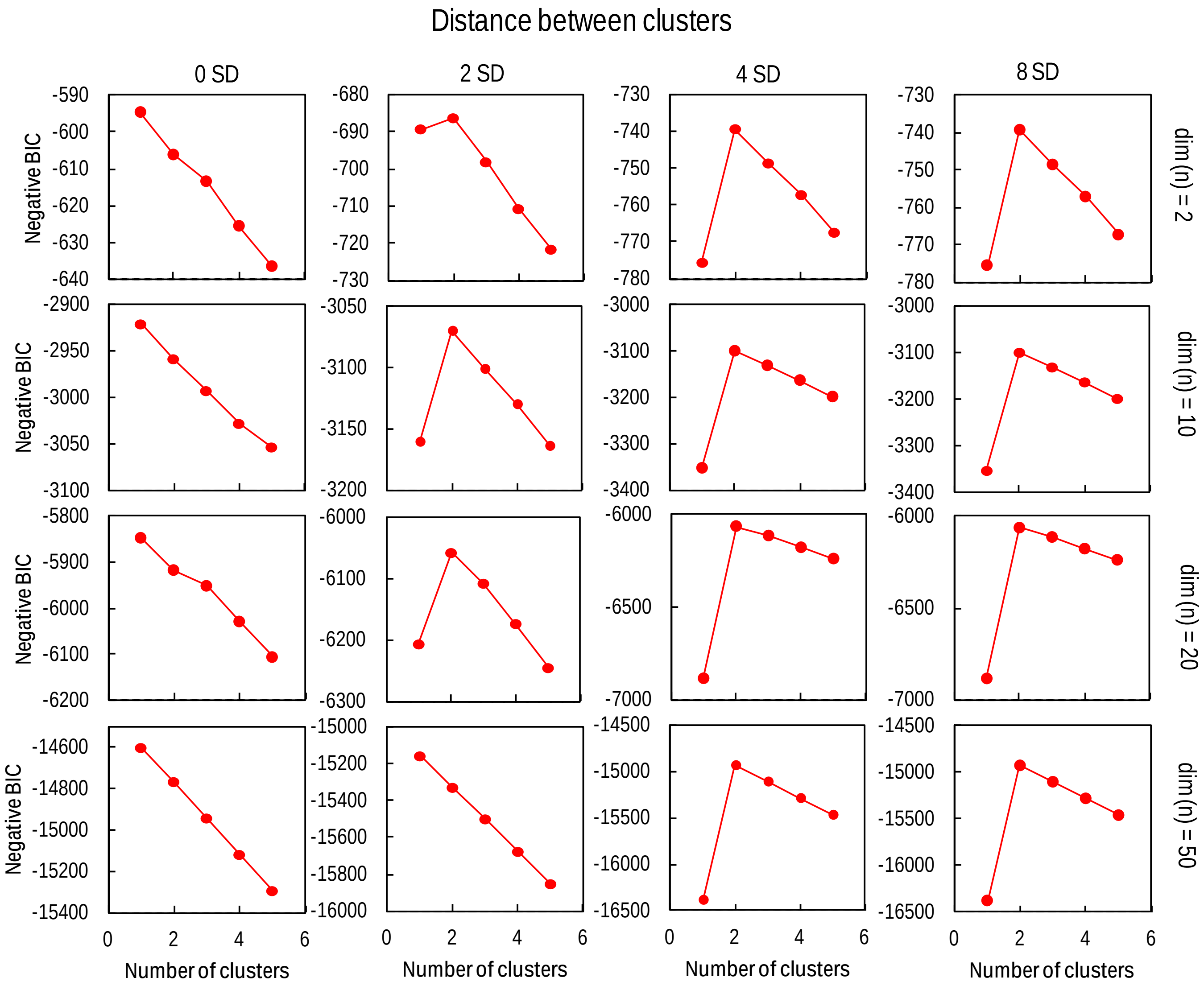

Supplementary Figure 2-4 | Negative BICs for various distances and dimensions, with N = 100.

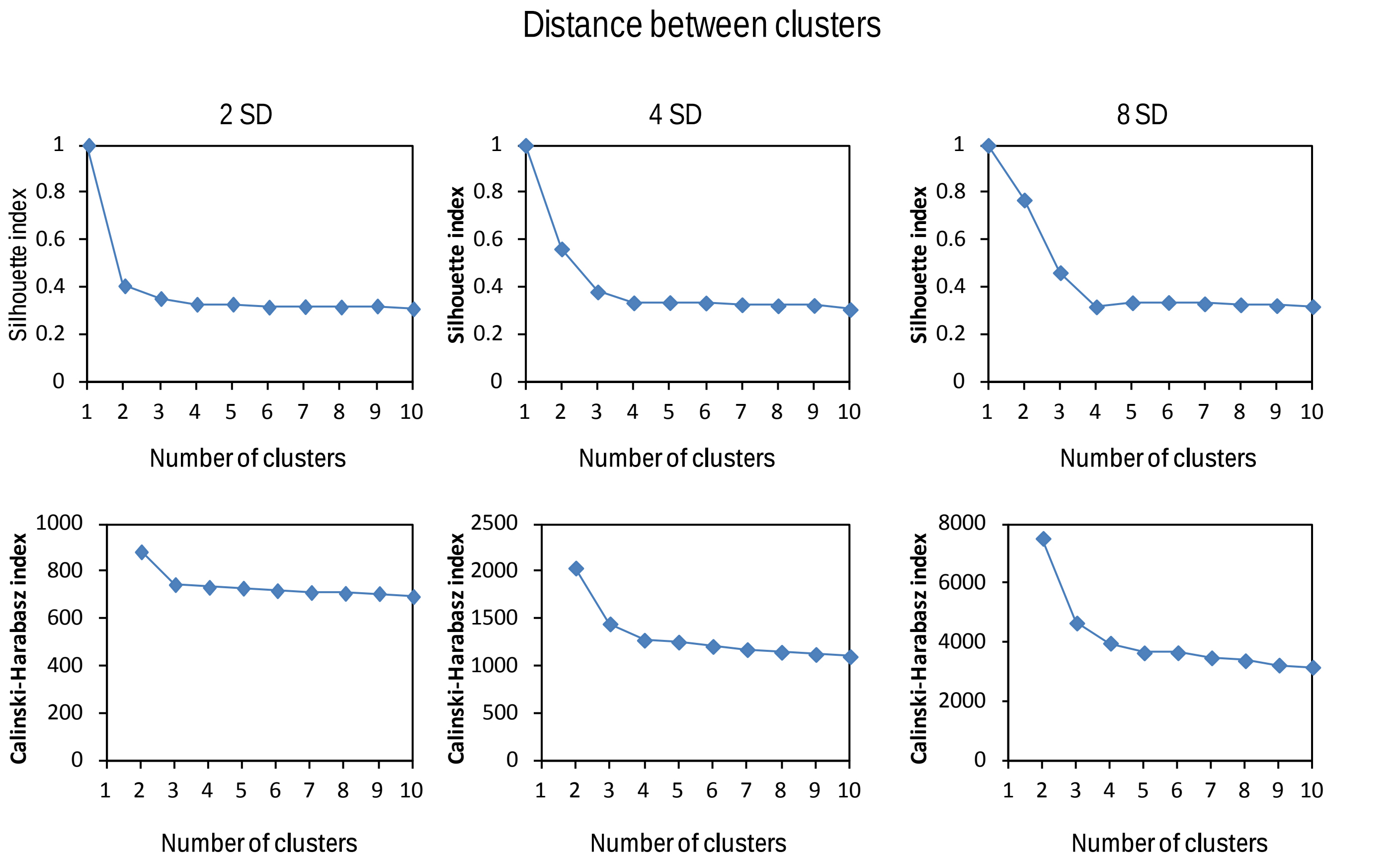

Supplementary Figure 2-5 | Silhouette index and Calinski-Harabasz indices for various distances, with N = 1000.

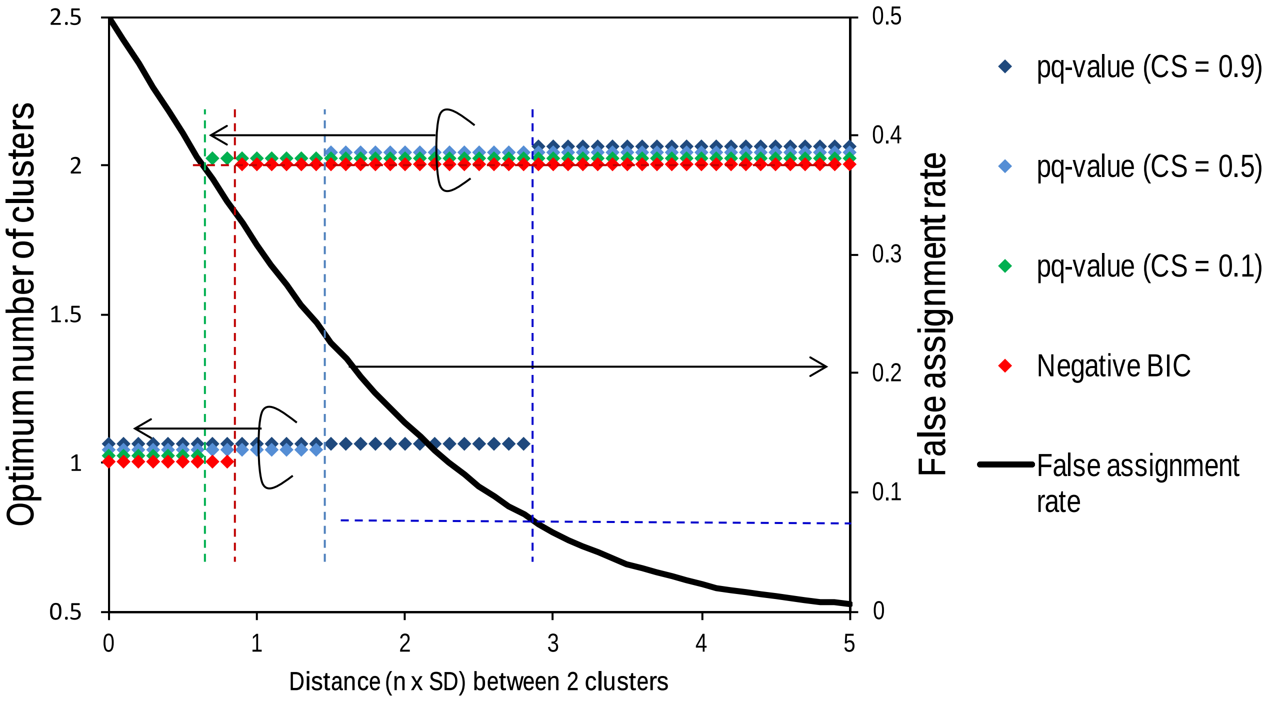

Supplementary Figure 3 | Comparison of optimum number of clusters between pq-value and negative BIC for one-dimensional data. As simplest case, two standard normal distributions placed with various distances (horizontal axis) were analyzed. Cross-over of optimum number of clusters in the negative BIC from one to two was occurred at short distance than that in pq-value while value of CS is 0.9. This behavior is consistent with that in Figure 4 d-f and Supplementary Figure 2-1, 2-2, 2-3, 2-4. As values of CS in the pq-value are decreased, the distances between clusters corresponding crossover points decrease.

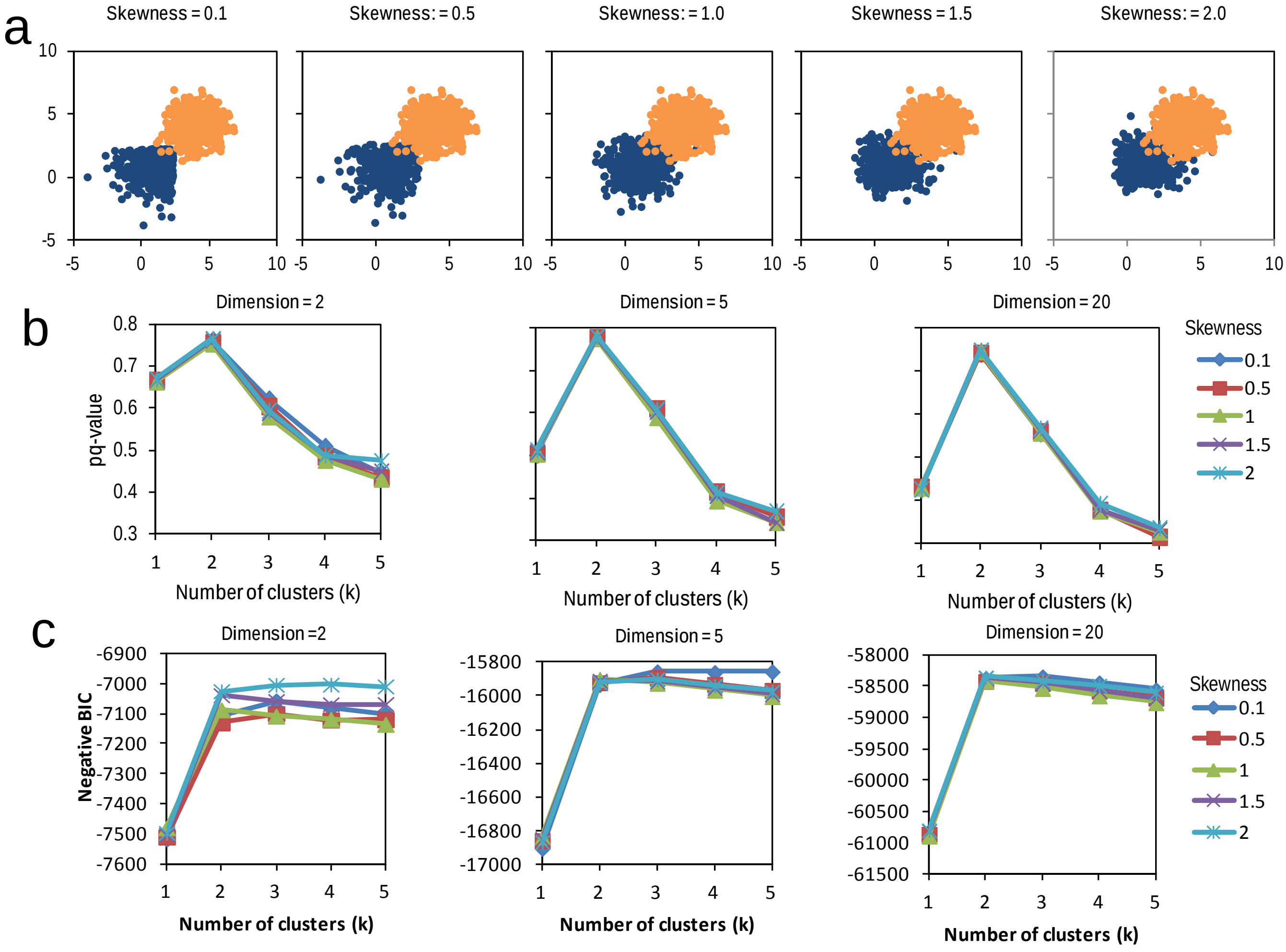

Supplementary Figure 4 | pq-values and negative BICs for generated data with various skewness levels. (a) Scatter plots of the generated data. Clusters represented by gray dots are skewed. (b) The optimum numbers of clusters obtained using pq-values are stable for all data sets in 2, 5 and 20 dimensions. (c) The optimum numbers of clusters obtained using negative BICs vary depending on the skewness levels in 2, 5 and 20 dimensions.

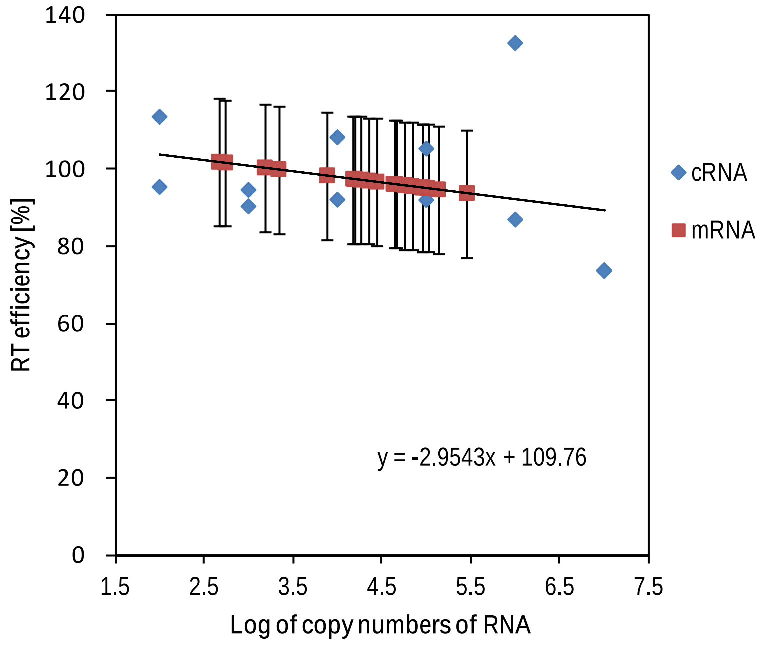

Supplementary Figure 5 | RT efficiency for calibrated mRNA by bead RT-qPCR of cRNA.

First, the copy number of cRNA was estimated by UV absorption measurements of the amount of purified, synthesized cRNA. Second, bead RT-qPCR was performed for the calibrated cRNA to determine the RT efficiency depending on the copy number of the cRNA. Finally, bead RT-qPCR was performed for the calibrated mRNA (measured in pg) to determine the copy number of mRNA relative to the RT efficiency.

Supplementary Table 1.| List of VFACs and numbers of cells

VFAC ID	Flow-cell ID	Sample ID	Sample type	Number of PMA(-) cells dispensed	Number of PMA(+) cells dispensed	Total number of cells dispensed
1	1	SN1-155	PMA-	50	0	50
2	1	SN1-156	Mix	50	50	100
3	1	SN1-157	PMA+	0	50	50
4	1	SN1-158	Mix	50	50	100
5	1	SN1-159	Mix	50	50	100
6	1	SN1-160	Mix	50	50	100
7	1	SN1-161	Mix	50	50	100
8	1	SN1-162	Mix	50	50	100
9	1	SN1-163	Mix	50	50	100
10	1	SN1-164	Mix	50	50	100
11	2	SN1-165	PMA+	0	70	70
12	2	SN1-166	Mix	35	35	70
13	2	SN1-167	PMA-	70	0	70
14	2	SN1-168	Mix	55	15	70
15	2	SN1-169	Mix	35	35	70
16	2	SN1-170	Mix	35	35	70
17	2	SN1-171	Mix	35	35	70
18	2	SN1-172	Mix	55	15	70
19	2	SN1-173	Mix	55	15	70
20	2	SN1-174	Mix	55	15	70
21	2	SN1-175	Mix	55	15	70
22	2	SN1-176	Mix	55	15	70
23	3	SN1-177	PMA-	70	0	70
24	3	SN1-178	Mix	55	15	70
25	3	SN1-179	PMA+	0	70	70
26	3	SN1-180	Mix	55	15	70
27	3	SN1-181	Mix	55	15	70
28	3	SN1-182	Mix	55	15	70
29	3	SN1-183	Mix	55	15	70
30	3	SN1-184	Mix	55	15	70
31	3	SN1-185	Mix	55	15	70
32	3	SN1-186	Mix	55	15	70
33	3	SN1-187	Mix	55	15	70
34	3	SN1-188	Mix	55	15	70

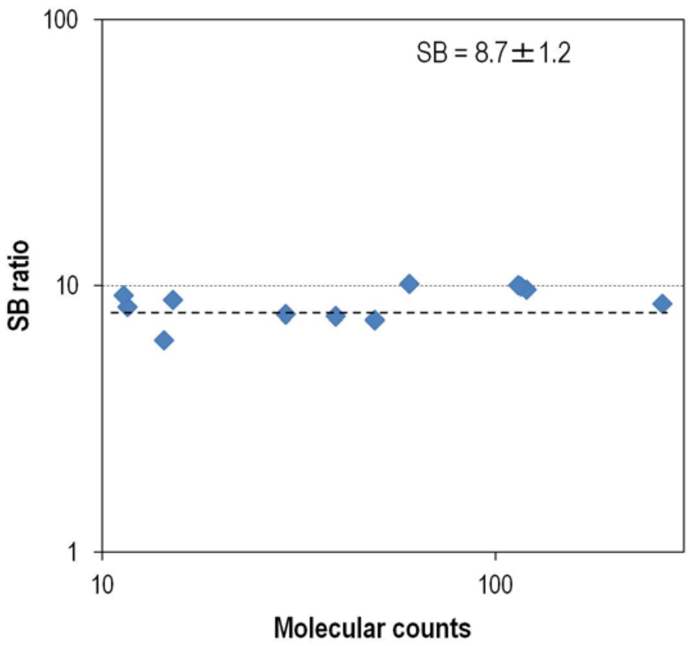

Supplementary Figure 6| Signal-to-background (SB) ratio.

The average SB ratio was determined for housekeeping genes with more than 15 copies (the detection limit).

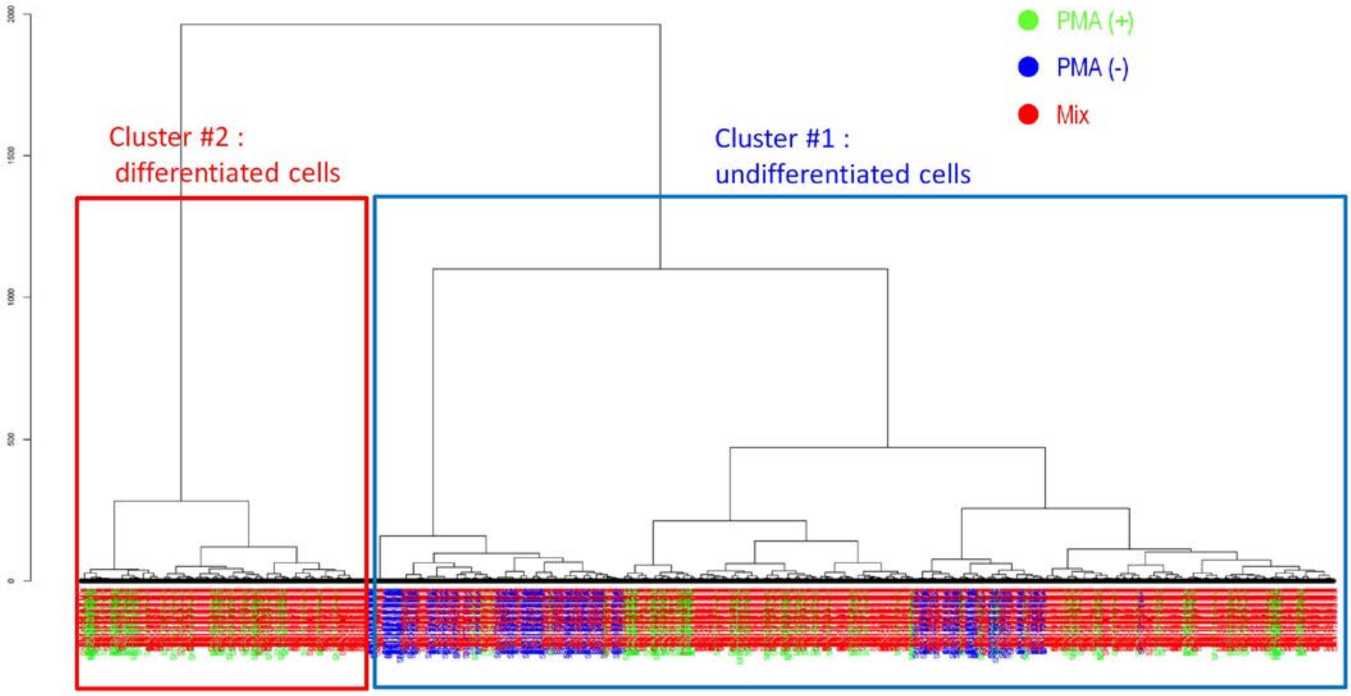

Supplementary Figure 7 | Dendrogram for 1967 cells with VFACs. Because the cluster of differentiated cells contains no PMA(-) cells, cluster #2 in the diagram corresponds to the cluster with red dots in the PCA visualization. In contrast, cluster #2 contains PMA(±) cells and a mixture of cells because PMA(+) cells include undifferentiated cells.

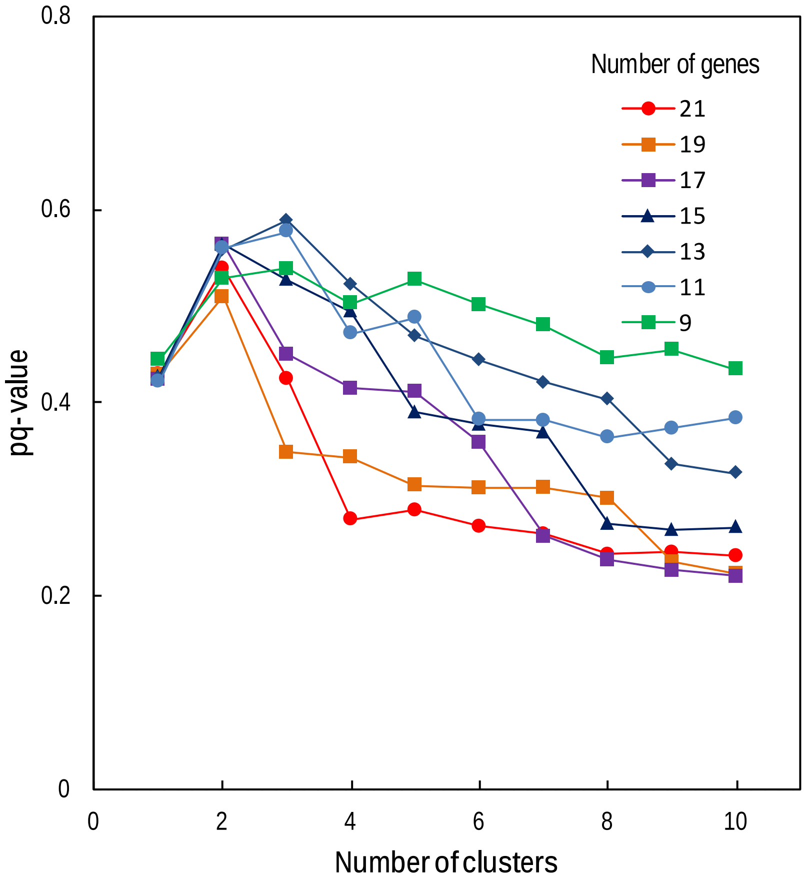

Supplementary Figure 8 | The pq-values for various numbers of genes.

The pq-value curves as a function of the number of clusters, as determined by selecting genes using a descending list of gene expression ratios of PMA(+) to PMA(-). Here, the parameters CS and SF for the pq-values were set to 0.9 and 1.0, respectively.

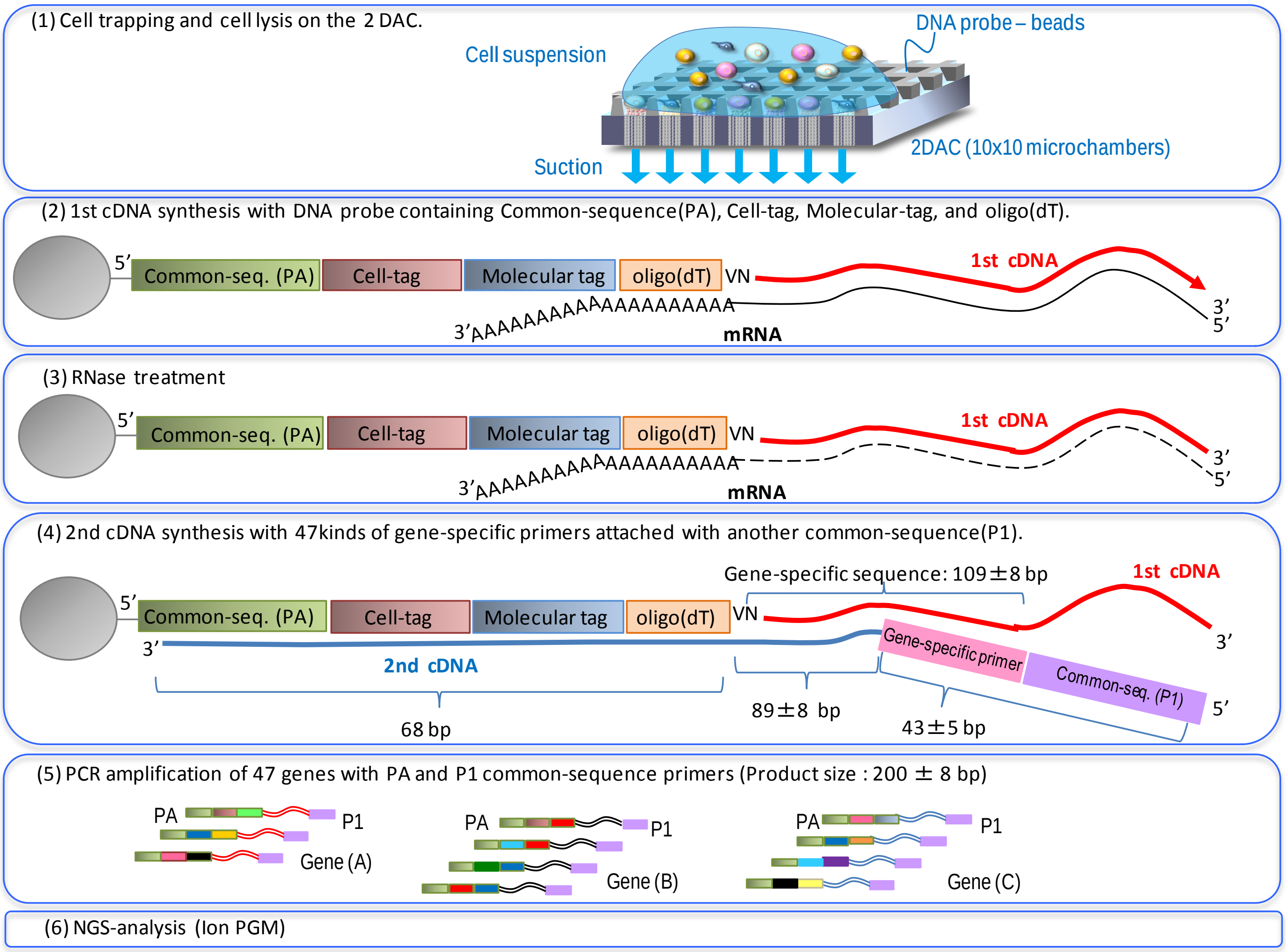

Supplementary Figure 9 | Schematic diagram of the processes. The lengths of the PCR products were adjusted to the sequencing length of Ion PGM. PCR products were obtained for 47 genes, including 20 housekeeping genes, 21 marker genes and 6 THP1-related genes (these 6 genes were not used for the analyses).

Supplementary Table 2 | Primer sequences for second cDNA synthesis.

No. 1-20: housekeeping genes; No. 21-41: differentiation marker genes; No. 42-47: THP1-related genes (not used for clustering analyses).

No.	Gene symbol	Primer name	Seq. (5'→3')	Length(bp)	Tm (℃)
1	ATP5B	P1_ATP5B	CCTCTCTATGGGCAGTCGGTGATCCCTAACCCAAAAAGCTTCATT	45	57.5
2	GAPDH	P1_GAPDH	CCTCTCTATGGGCAGTCGGTGATCACTGAATCTCCCCTCCTCACA	45	59.3
3	GUSB	P1_GUSB	CCTCTCTATGGGCAGTCGGTGATCGTTTCTGGCCTGGGTTTTG	43	59.6
4	HMBS	P1_HMBS	CCTCTCTATGGGCAGTCGGTGATGATGACTGCCTTGCCTCCTC	43	60.3
5	HPRT1	P1_HPRT1	CCTCTCTATGGGCAGTCGGTGATTAGTAGTGTTTCAGTAATGTTGACT	48	55.8
6	RPL4	P1_RPL4	CCTCTCTATGGGCAGTCGGTGATAAGAAGCCTGCTGCATAAAC	43	58
7	RPLP1	P1_RPLP1	CCTCTCTATGGGCAGTCGGTGATAAGTGGAAGCAAAGAAAGAAGAATCC	49	59.9
8	RPS18	P1_RPS18	CCTCTCTATGGGCAGTCGGTGATGTGTCCGAGGCCAGCACA	41	58.5
9	RPL13A	P1_RPL13A	CCTCTCTATGGGCAGTCGGTGATTCTAGAAGCAGAAATAGACTGGGAA	48	57.5
10	RPS20	P1_RPS20	CCTCTCTATGGGCAGTCGGTGATGAGATTGTTAAGCAGATTACTTCCA	48	56.7
11	ALDOA	P1_ALDOA	CCTCTCTATGGGCAGTCGGTGATTTGCCCGCGCTCTTTCTTC	42	59.7
12	B2M	P1_B2M	CCTCTCTATGGGCAGTCGGTGATATTCATATTTACTTCTTATACATTTGA	50	51.9
13	EEF1G	P1_EEF1G	CCTCTCTATGGGCAGTCGGTGATAAAGCCTTCAATCAGGGCAA	43	57.4
14	SDHA	P1_SDHA	CCTCTCTATGGGCAGTCGGTGATCCAGGGAGCGTGGCACTT	41	62.2
15	TBP	P1_TBP	CCTCTCTATGGGCAGTCGGTGATCTCCAGTATTGCAGGACAGA	43	57.7
16	VIM	P1_VIM	CCTCTCTATGGGCAGTCGGTGATAATCTTGTGCTAGAATACTT	43	51.2
17	RPLP0	P1_RPLP0	CCTCTCTATGGGCAGTCGGTGATTCGGACGAGGATATGGGATT	43	59
18	RPLP2	P1_RPLP2	CCTCTCTATGGGCAGTCGGTGATATGAGAAGAAGGAGGAGTCTG	44	57.6
19	RPLP27	P1_RPLP27	CCTCTCTATGGGCAGTCGGTGATGGGAGGCCAAGGTCAAGT	41	59.2
20	OAZ1	P1_OAZ1	CCTCTCTATGGGCAGTCGGTGATAGAAGTTTCTTATTTGGAGTCT	45	54.4
21	PTGS1	P1_PTGS1	CCTCTCTATGGGCAGTCGGTGATATAGCCATCGACTCCTGCCT	43	62.3
22	GSN	P1_GSN	CCTCTCTATGGGCAGTCGGTGATTTTTACAGTATCCAAAAATAGCCCTGC	50	59.3
23	HOPX	P1_HOPX	CCTCTCTATGGGCAGTCGGTGATAAAACCACCAAAGTTCTTGAATTTACT	50	58.4
24	IL1RN	P1_IL1RN	CCTCTCTATGGGCAGTCGGTGATAGCACCTAGCCTCGCTCTTG	43	60.7
25	CCL4L2	P1_CCL4L2	CCTCTCTATGGGCAGTCGGTGATATGGGGATGGTCCACTCTCAC	44	62.2
26	ITGB2	P1_ITGB2	CCTCTCTATGGGCAGTCGGTGATAAAGGTGGTGCCAATTTATTTACATTT	50	58.4
27	MYO1E	P1_MYO1E	CCTCTCTATGGGCAGTCGGTGATAGAGGCCTGGAACAGATTCTCC	45	61.7
28	POU6F1	P1_POU6F1	CCTCTCTATGGGCAGTCGGTGATACTGTTCCCAACACAGACAAAGC	46	61.7
29	CD109	P1_CD109	CCTCTCTATGGGCAGTCGGTGATATTGATCAAGGTGATTCTGAAAGTTTTA	51	57.5
30	CD53	P1_CD53	CCTCTCTATGGGCAGTCGGTGATCTGATAATCACAGTAAGAAGACTTCTGGT	52	59.4
31	TMEM158	P1_TMEM158	CCTCTCTATGGGCAGTCGGTGATACCGCTGAAAATGAACTTTATGAACC	49	60.4
32	IF116	P1_IF116	CCTCTCTATGGGCAGTCGGTGATGAATGGGGTATTGGGAGTGCTTT	46	61.3
33	APOC4	P1_APOC4	CCTCTCTATGGGCAGTCGGTGATTTGAGCCCAGGAGTTCGAGAC	44	60.3
34	STAMBPL1	P1_STAMBPL1	CCTCTCTATGGGCAGTCGGTGATGTTGCTCTGTGTCAAGAGAGGT	45	60.5
35	CD36	P1_CD36	CCTCTCTATGGGCAGTCGGTGATAACCTATAAATATTATCACGCAGATCACT	52	58.5
36	APOC1	P1_APOC1	CCTCTCTATGGGCAGTCGGTGATACCTGAAGGGTGACATCCCAG	44	60.6
37	CRIP1	P1_CRIP1	CCTCTCTATGGGCAGTCGGTGATGTGGTGGAGACCCCATCCTTG	44	59.8
38	GNB2L1	P1_GNB2L1	CCTCTCTATGGGCAGTCGGTGATAGACTCTGTTTGCTGGCTACAC	45	60
39	CCL5	P1_CCL5	CCTCTCTATGGGCAGTCGGTGATTAGAGGCAAGGAGGGGAGGAAC	45	61.6

40	EMP1	P1_EMP1	CCTCTCTATGGGCAGTCGGTGATCTAGTACTGTATTGGGCTTCTTCGT	48	61.3
41	CHAC1	P1_CHAC1	CCTCTCTATGGGCAGTCGGTGATGCCACTCCTGCCTGTGTGTTG	44	61.1
42	SH3GRL3	P1_SH3GRL3	CCTCTCTATGGGCAGTCGGTGATGGCTGATGGGCACCTCTGTTG	44	61.1
43	FABP3	P1_FABP3	CCTCTCTATGGGCAGTCGGTGATGTCACTTCAGGCCCATCCTAC	44	60
44	ELL2	P1_ELL2	CCTCTCTATGGGCAGTCGGTGATACCGTGTTAGGACTCTCAAAGGTA	47	61.4
45	MMP14	P1_MMP14	CCTCTCTATGGGCAGTCGGTGATGGGGTAGGGGAAATGGGGTGAA	45	60.4
46	PDGFA	P1_PDGFA	CCTCTCTATGGGCAGTCGGTGATGTAAAGGTGAAGTTTGTATGTTTACCTA	51	56.8
47	SPP1	P1_SPP1	CCTCTCTATGGGCAGTCGGTGATTGTTGTGATTATCTTTTTGTGGTGTGAA	51	59.7

Supplementary Table 3 | PCR primer sequences.

No.	Primer name	Seq. (5'→3')
1	5' side of PA	CCATCTCATCCCTGCGTGTCT
2	5' side of P1	CCTCTCTATGGGCAGTCGG

Supplementary Table 4 | RT probe sequences. The common sequence (Tag(A), 30 nt) used for the PCR amplification process is shown in red; the cell tag (7 nt, 100 sequences) that enables cell distinction is shown in blue; and the molecular tag (7 nt) that enables molecular distinction for the revision of PCR-bias is shown in green.

No.	Prove name	Seq. (5'→3')	5' modification
1	PA_cell tag1_mol tag_(dT)18VN	CCATCTCATCCCTGCGTGTCTCCGACTCAGTCGCGTANNNNNNNTTTTTTTTTTTTTTTTTTVN	C6_dual Biotin
2	PA_cell tag2_mol tag_(dT)18VN	CCATCTCATCCCTGCGTGTCTCCGACTCAGTATCGCGNNNNNNNTTTTTTTTTTTTTTTTTTVN	C6_dual Biotin
3	PA_cell tag3_mol tag_(dT)18VN	CCATCTCATCCCTGCGTGTCTCCGACTCAGCGCGATANNNNNNNTTTTTTTTTTTTTTTTTTVN	C6_dual Biotin
4	PA_cell tag4_mol tag_(dT)18VN	CCATCTCATCCCTGCGTGTCTCCGACTCAGATACGCGNNNNNNNTTTTTTTTTTTTTTTTTTVN	C6_dual Biotin
5	PA_cell tag5_mol tag_(dT)18VN	CCATCTCATCCCTGCGTGTCTCCGACTCAGTCGTACGNNNNNNNTTTTTTTTTTTTTTTTTTVN	C6_dual Biotin
6	PA_cell tag6_mol tag_(dT)18VN	CCATCTCATCCCTGCGTGTCTCCGACTCAGACGCGTANNNNNNNTTTTTTTTTTTTTTTTTTVN	C6_dual Biotin
7	PA_cell tag7_mol tag_(dT)18VN	CCATCTCATCCCTGCGTGTCTCCGACTCAGTCGATCGNNNNNNNTTTTTTTTTTTTTTTTTTVN	C6_dual Biotin
8	PA_cell tag8_mol tag_(dT)18VN	CCATCTCATCCCTGCGTGTCTCCGACTCAGACGATCGNNNNNNNTTTTTTTTTTTTTTTTTTVN	C6_dual Biotin
9	PA_cell tag9_mol tag_(dT)18VN	CCATCTCATCCCTGCGTGTCTCCGACTCAGACGTACGNNNNNNNTTTTTTTTTTTTTTTTTTVN	C6_dual Biotin
10	PA_cell tag10_mol tag_(dT)18VN	CCATCTCATCCCTGCGTGTCTCCGACTCAGCGATACGNNNNNNNTTTTTTTTTTTTTTTTTTVN	C6_dual Biotin
11	PA_cell tag11_mol tag_(dT)18VN	CCATCTCATCCCTGCGTGTCTCCGACTCAGCGTATCGNNNNNNNTTTTTTTTTTTTTTTTTTVN	C6_dual Biotin
12	PA_cell tag12_mol tag_(dT)18VN	CCATCTCATCCCTGCGTGTCTCCGACTCAGCGTACGCNNNNNNNTTTTTTTTTTTTTTTTTTVN	C6_dual Biotin
13	PA_cell tag13_mol tag_(dT)18VN	CCATCTCATCCCTGCGTGTCTCCGACTCAGGCGTACGNNNNNNNTTTTTTTTTTTTTTTTTTVN	C6_dual Biotin
14	PA_cell tag14_mol tag_(dT)18VN	CCATCTCATCCCTGCGTGTCTCCGACTCAGTACGTCGNNNNNNNTTTTTTTTTTTTTTTTTTVN	C6_dual Biotin
15	PA_cell tag15_mol tag_(dT)18VN	CCATCTCATCCCTGCGTGTCTCCGACTCAGCGACGTANNNNNNNTTTTTTTTTTTTTTTTTTVN	C6_dual Biotin
16	PA_cell tag16_mol tag_(dT)18VN	CCATCTCATCCCTGCGTGTCTCCGACTCAGCGCGCTANNNNNNNTTTTTTTTTTTTTTTTTTVN	C6_dual Biotin
17	PA_cell tag17_mol tag_(dT)18VN	CCATCTCATCCCTGCGTGTCTCCGACTCAGTAGCGCGNNNNNNNTTTTTTTTTTTTTTTTTTVN	C6_dual Biotin
18	PA_cell tag18_mol tag_(dT)18VN	CCATCTCATCCCTGCGTGTCTCCGACTCAGACTAGCGNNNNNNNTTTTTTTTTTTTTTTTTTVN	C6_dual Biotin
19	PA_cell tag19_mol tag_(dT)18VN	CCATCTCATCCCTGCGTGTCTCCGACTCAGCGATCGCNNNNNNNTTTTTTTTTTTTTTTTTTVN	C6_dual Biotin
20	PA_cell tag20_mol tag_(dT)18VN	CCATCTCATCCCTGCGTGTCTCCGACTCAGGCGATCGNNNNNNNTTTTTTTTTTTTTTTTTTVN	C6_dual Biotin
21	PA_cell tag21_mol tag_(dT)18VN	CCATCTCATCCCTGCGTGTCTCCGACTCAGGTACGCGNNNNNNNTTTTTTTTTTTTTTTTTTVN	C6_dual Biotin
22	PA_cell tag22_mol tag_(dT)18VN	CCATCTCATCCCTGCGTGTCTCCGACTCAGTACGCGCNNNNNNNTTTTTTTTTTTTTTTTTTVN	C6_dual Biotin
23	PA_cell tag23_mol tag_(dT)18VN	CCATCTCATCCCTGCGTGTCTCCGACTCAGGCGCGTANNNNNNNTTTTTTTTTTTTTTTTTTVN	C6_dual Biotin
24	PA_cell tag24_mol tag_(dT)18VN	CCATCTCATCCCTGCGTGTCTCCGACTCAGTATGCGCNNNNNNNTTTTTTTTTTTTTTTTTTVN	C6_dual Biotin
25	PA_cell tag25_mol tag_(dT)18VN	CCATCTCATCCCTGCGTGTCTCCGACTCAGGCGCATANNNNNNNTTTTTTTTTTTTTTTTTTVN	C6_dual Biotin
26	PA_cell tag26_mol tag_(dT)18VN	CCATCTCATCCCTGCGTGTCTCCGACTCAGCTAGTCGNNNNNNNTTTTTTTTTTTTTTTTTTVN	C6_dual Biotin
27	PA_cell tag27_mol tag_(dT)18VN	CCATCTCATCCCTGCGTGTCTCCGACTCAGGTCGACGNNNNNNNTTTTTTTTTTTTTTTTTTVN	C6_dual Biotin
28	PA_cell tag28_mol tag_(dT)18VN	CCATCTCATCCCTGCGTGTCTCCGACTCAGTAGTGCGNNNNNNNTTTTTTTTTTTTTTTTTTVN	C6_dual Biotin
29	PA_cell tag29_mol tag_(dT)18VN	CCATCTCATCCCTGCGTGTCTCCGACTCAGCGCACTANNNNNNNTTTTTTTTTTTTTTTTTTVN	C6_dual Biotin
30	PA_cell tag30_mol tag_(dT)18VN	CCATCTCATCCCTGCGTGTCTCCGACTCAGTACGACGNNNNNNNTTTTTTTTTTTTTTTTTTVN	C6_dual Biotin
31	PA_cell tag31_mol tag_(dT)18VN	CCATCTCATCCCTGCGTGTCTCCGACTCAGCGTCGTANNNNNNNTTTTTTTTTTTTTTTTTTVN	C6_dual Biotin
32	PA_cell tag32_mol tag_(dT)18VN	CCATCTCATCCCTGCGTGTCTCCGACTCAGTCTAGCGNNNNNNNTTTTTTTTTTTTTTTTTTVN	C6_dual Biotin
33	PA_cell tag33_mol tag_(dT)18VN	CCATCTCATCCCTGCGTGTCTCCGACTCAGGTACGCTNNNNNNNTTTTTTTTTTTTTTTTTTVN	C6_dual Biotin
34	PA_cell tag34_mol tag_(dT)18VN	CCATCTCATCCCTGCGTGTCTCCGACTCAGTCGCACGNNNNNNNTTTTTTTTTTTTTTTTTTVN	C6_dual Biotin
35	PA_cell tag35_mol tag_(dT)18VN	CCATCTCATCCCTGCGTGTCTCCGACTCAGGTATGCGNNNNNNNTTTTTTTTTTTTTTTTTTVN	C6_dual Biotin
36	PA_cell tag36_mol tag_(dT)18VN	CCATCTCATCCCTGCGTGTCTCCGACTCAGATCGTCGNNNNNNNTTTTTTTTTTTTTTTTTTVN	C6_dual Biotin
37	PA_cell tag37_mol tag_(dT)18VN	CCATCTCATCCCTGCGTGTCTCCGACTCAGGTCGCTANNNNNNNTTTTTTTTTTTTTTTTTTVN	C6_dual Biotin
38	PA_cell tag38_mol tag_(dT)18VN	CCATCTCATCCCTGCGTGTCTCCGACTCAGCTACGCGNNNNNNNTTTTTTTTTTTTTTTTTTVN	C6_dual Biotin

No.	Prove name	Seq. (5'→3')	5' modification
39	PA_cell tag39_mol tag_(dT)18VN	CCATCTCATCCCTGCGTGTCTCCGACTCAGGATACGCNNNNNNNTTTTTTTTTTTTTTTTTTVN	C6_dual Biotin
40	PA_cell tag40_mol tag_(dT)18VN	CCATCTCATCCCTGCGTGTCTCCGACTCAGGCTAGCGNNNNNNNTTTTTTTTTTTTTTTTTTVN	C6_dual Biotin
41	PA_cell tag41_mol tag_(dT)18VN	CCATCTCATCCCTGCGTGTCTCCGACTCAGCGCTAGCNNNNNNNTTTTTTTTTTTTTTTTTTVN	C6_dual Biotin
42	PA_cell tag42_mol tag_(dT)18VN	CCATCTCATCCCTGCGTGTCTCCGACTCAGTAGTCGCNNNNNNNTTTTTTTTTTTTTTTTTTVN	C6_dual Biotin
43	PA_cell tag43_mol tag_(dT)18VN	CCATCTCATCCCTGCGTGTCTCCGACTCAGGCGACTANNNNNNNTTTTTTTTTTTTTTTTTTVN	C6_dual Biotin
44	PA_cell tag44_mol tag_(dT)18VN	CCATCTCATCCCTGCGTGTCTCCGACTCAGGTGCGTANNNNNNNTTTTTTTTTTTTTTTTTTVN	C6_dual Biotin
45	PA_cell tag45_mol tag_(dT)18VN	CCATCTCATCCCTGCGTGTCTCCGACTCAGATCGACGNNNNNNNTTTTTTTTTTTTTTTTTTVN	C6_dual Biotin
46	PA_cell tag46_mol tag_(dT)18VN	CCATCTCATCCCTGCGTGTCTCCGACTCAGCTAGCGCNNNNNNNTTTTTTTTTTTTTTTTTTVN	C6_dual Biotin
47	PA_cell tag47_mol tag_(dT)18VN	CCATCTCATCCCTGCGTGTCTCCGACTCAGGAGCGTANNNNNNNTTTTTTTTTTTTTTTTTTVN	C6_dual Biotin
48	PA_cell tag48_mol tag_(dT)18VN	CCATCTCATCCCTGCGTGTCTCCGACTCAGCTATCGCNNNNNNNTTTTTTTTTTTTTTTTTTVN	C6_dual Biotin
49	PA_cell tag49_mol tag_(dT)18VN	CCATCTCATCCCTGCGTGTCTCCGACTCAGCGATAGCNNNNNNNTTTTTTTTTTTTTTTTTTVN	C6_dual Biotin
50	PA_cell tag50_mol tag_(dT)18VN	CCATCTCATCCCTGCGTGTCTCCGACTCAGGCTATCGNNNNNNNTTTTTTTTTTTTTTTTTTVN	C6_dual Biotin
51	PA_cell tag51_mol tag_(dT)18VN	CCATCTCATCCCTGCGTGTCTCCGACTCAGGTATCGCNNNNNNNTTTTTTTTTTTTTTTTTTVN	C6_dual Biotin
52	PA_cell tag52_mol tag_(dT)18VN	CCATCTCATCCCTGCGTGTCTCCGACTCAGATGCGCGNNNNNNNTTTTTTTTTTTTTTTTTTVN	C6_dual Biotin
53	PA_cell tag53_mol tag_(dT)18VN	CCATCTCATCCCTGCGTGTCTCCGACTCAGTCGACGCNNNNNNNTTTTTTTTTTTTTTTTTTVN	C6_dual Biotin
54	PA_cell tag54_mol tag_(dT)18VN	CCATCTCATCCCTGCGTGTCTCCGACTCAGTGTCGCGNNNNNNNTTTTTTTTTTTTTTTTTTVN	C6_dual Biotin
55	PA_cell tag55_mol tag_(dT)18VN	CCATCTCATCCCTGCGTGTCTCCGACTCAGGACGCTANNNNNNNTTTTTTTTTTTTTTTTTTVN	C6_dual Biotin
56	PA_cell tag56_mol tag_(dT)18VN	CCATCTCATCCCTGCGTGTCTCCGACTCAGCGTAGCGNNNNNNNTTTTTTTTTTTTTTTTTTVN	C6_dual Biotin
57	PA_cell tag57_mol tag_(dT)18VN	CCATCTCATCCCTGCGTGTCTCCGACTCAGCGCTACGNNNNNNNTTTTTTTTTTTTTTTTTTVN	C6_dual Biotin
58	PA_cell tag58_mol tag_(dT)18VN	CCATCTCATCCCTGCGTGTCTCCGACTCAGCTAGACGNNNNNNNTTTTTTTTTTTTTTTTTTVN	C6_dual Biotin
59	PA_cell tag59_mol tag_(dT)18VN	CCATCTCATCCCTGCGTGTCTCCGACTCAGCTATGCGNNNNNNNTTTTTTTTTTTTTTTTTTVN	C6_dual Biotin
60	PA_cell tag60_mol tag_(dT)18VN	CCATCTCATCCCTGCGTGTCTCCGACTCAGAGTCGCGNNNNNNNTTTTTTTTTTTTTTTTTTVN	C6_dual Biotin
61	PA_cell tag61_mol tag_(dT)18VN	CCATCTCATCCCTGCGTGTCTCCGACTCAGCGTATGCNNNNNNNTTTTTTTTTTTTTTTTTTVN	C6_dual Biotin
62	PA_cell tag62_mol tag_(dT)18VN	CCATCTCATCCCTGCGTGTCTCCGACTCAGGCATACGNNNNNNNTTTTTTTTTTTTTTTTTTVN	C6_dual Biotin
63	PA_cell tag63_mol tag_(dT)18VN	CCATCTCATCCCTGCGTGTCTCCGACTCAGTCGCGCGNNNNNNNTTTTTTTTTTTTTTTTTTVN	C6_dual Biotin
64	PA_cell tag64_mol tag_(dT)18VN	CCATCTCATCCCTGCGTGTCTCCGACTCAGCATACGCNNNNNNNTTTTTTTTTTTTTTTTTTVN	C6_dual Biotin
65	PA_cell tag65_mol tag_(dT)18VN	CCATCTCATCCCTGCGTGTCTCCGACTCAGAGTACGCNNNNNNNTTTTTTTTTTTTTTTTTTVN	C6_dual Biotin
66	PA_cell tag66_mol tag_(dT)18VN	CCATCTCATCCCTGCGTGTCTCCGACTCAGTCACGCGNNNNNNNTTTTTTTTTTTTTTTTTTVN	C6_dual Biotin
67	PA_cell tag67_mol tag_(dT)18VN	CCATCTCATCCCTGCGTGTCTCCGACTCAGACTACGCNNNNNNNTTTTTTTTTTTTTTTTTTVN	C6_dual Biotin
68	PA_cell tag68_mol tag_(dT)18VN	CCATCTCATCCCTGCGTGTCTCCGACTCAGATAGCGCNNNNNNNTTTTTTTTTTTTTTTTTTVN	C6_dual Biotin
69	PA_cell tag69_mol tag_(dT)18VN	CCATCTCATCCCTGCGTGTCTCCGACTCAGTAGCACGNNNNNNNTTTTTTTTTTTTTTTTTTVN	C6_dual Biotin
70	PA_cell tag70_mol tag_(dT)18VN	CCATCTCATCCCTGCGTGTCTCCGACTCAGCGTGCTANNNNNNNTTTTTTTTTTTTTTTTTTVN	C6_dual Biotin
71	PA_cell tag71_mol tag_(dT)18VN	CCATCTCATCCCTGCGTGTCTCCGACTCAGCACGCTANNNNNNNTTTTTTTTTTTTTTTTTTVN	C6_dual Biotin
72	PA_cell tag72_mol tag_(dT)18VN	CCATCTCATCCCTGCGTGTCTCCGACTCAGGATAGCGNNNNNNNTTTTTTTTTTTTTTTTTTVN	C6_dual Biotin
73	PA_cell tag73_mol tag_(dT)18VN	CCATCTCATCCCTGCGTGTCTCCGACTCAGACGCTCGNNNNNNNTTTTTTTTTTTTTTTTTTVN	C6_dual Biotin
74	PA_cell tag74_mol tag_(dT)18VN	CCATCTCATCCCTGCGTGTCTCCGACTCAGTCGTGCGNNNNNNNTTTTTTTTTTTTTTTTTTVN	C6_dual Biotin
75	PA_cell tag75_mol tag_(dT)18VN	CCATCTCATCCCTGCGTGTCTCCGACTCAGACTCGCGNNNNNNNTTTTTTTTTTTTTTTTTTVN	C6_dual Biotin
76	PA_cell tag76_mol tag_(dT)18VN	CCATCTCATCCCTGCGTGTCTCCGACTCAGAGCGCTANNNNNNNTTTTTTTTTTTTTTTTTTVN	C6_dual Biotin
77	PA_cell tag77_mol tag_(dT)18VN	CCATCTCATCCCTGCGTGTCTCCGACTCAGTGACGCGNNNNNNNTTTTTTTTTTTTTTTTTTVN	C6_dual Biotin
78	PA_cell tag78_mol tag_(dT)18VN	CCATCTCATCCCTGCGTGTCTCCGACTCAGTGCGTCGNNNNNNNTTTTTTTTTTTTTTTTTTVN	C6_dual Biotin
79	PA_cell ta79_mol tag_(dT)18VN	CCATCTCATCCCTGCGTGTCTCCGACTCAGCTCGCTANNNNNNNTTTTTTTTTTTTTTTTTTVN	C6_dual Biotin
80	PA_cell tag80_mol tag_(dT)18VN	CCATCTCATCCCTGCGTGTCTCCGACTCAGTGCGCTANNNNNNNTTTTTTTTTTTTTTTTTTVN	C6_dual Biotin

No.	Prove name	Seq. (5'→3')	5' modification
81	PA_cell tag81_mol tag_(dT)18VN	CCATCTCATCCCTGCGTGTCTCCGACTCAGTAGACGCNNNNNNNTTTTTTTTTTTTTTTTTTVN	C6_dual Biotin
82	PA_cell tag82_mol tag_(dT)18VN	CCATCTCATCCCTGCGTGTCTCCGACTCAGGCGTCTANNNNNNNTTTTTTTTTTTTTTTTTTVN	C6_dual Biotin
83	PA_cell tag83_mol tag_(dT)18VN	CCATCTCATCCCTGCGTGTCTCCGACTCAGTAGCTCGNNNNNNNTTTTTTTTTTTTTTTTTTVN	C6_dual Biotin
84	PA_cell tag84_mol tag_(dT)18VN	CCATCTCATCCCTGCGTGTCTCCGACTCAGCGAGCTANNNNNNNTTTTTTTTTTTTTTTTTTVN	C6_dual Biotin
85	PA_cell tag85mol tag_(dT)18VN	CCATCTCATCCCTGCGTGTCTCCGACTCAGCTCGACGNNNNNNNTTTTTTTTTTTTTTTTTTVN	C6_dual Biotin
86	PA_cell tag86_mol tag_(dT)18VN	CCATCTCATCCCTGCGTGTCTCCGACTCAGTGTACGCNNNNNNNTTTTTTTTTTTTTTTTTTVN	C6_dual Biotin
87	PA_cell tag87_mol tag_(dT)18VN	CCATCTCATCCCTGCGTGTCTCCGACTCAGTGCGACGNNNNNNNTTTTTTTTTTTTTTTTTTVN	C6_dual Biotin
88	PA_cell tag88_mol tag_(dT)18VN	CCATCTCATCCCTGCGTGTCTCCGACTCAGTCGAGCGNNNNNNNTTTTTTTTTTTTTTTTTTVN	C6_dual Biotin
89	PA_cell tag89_mol tag_(dT)18VN	CCATCTCATCCCTGCGTGTCTCCGACTCAGCGCGACGNNNNNNNTTTTTTTTTTTTTTTTTTVN	C6_dual Biotin
90	PA_cell tag90_mol tag_(dT)18VN	CCATCTCATCCCTGCGTGTCTCCGACTCAGCGTCGCGNNNNNNNTTTTTTTTTTTTTTTTTTVN	C6_dual Biotin
91	PA_cell tag91_mol tag_(dT)18VN	CCATCTCATCCCTGCGTGTCTCCGACTCAGACGCACGNNNNNNNTTTTTTTTTTTTTTTTTTVN	C6_dual Biotin
92	PA_cell tag92_mol tag_(dT)18VN	CCATCTCATCCCTGCGTGTCTCCGACTCAGCGACGCGNNNNNNNTTTTTTTTTTTTTTTTTTVN	C6_dual Biotin
93	PA_cell tag93_mol tag_(dT)18VN	CCATCTCATCCCTGCGTGTCTCCGACTCAGCGCGTCGNNNNNNNTTTTTTTTTTTTTTTTTTVN	C6_dual Biotin
94	PA_cell tag94_mol tag_(dT)18VN	CCATCTCATCCCTGCGTGTCTCCGACTCAGACGACGCNNNNNNNTTTTTTTTTTTTTTTTTTVN	C6_dual Biotin
95	PA_cell tag95_mol tag_(dT)18VN	CCATCTCATCCCTGCGTGTCTCCGACTCAGATCGCGCNNNNNNNTTTTTTTTTTTTTTTTTTVN	C6_dual Biotin
96	PA_cell tag96_mol tag_(dT)18VN	CCATCTCATCCCTGCGTGTCTCCGACTCAGGATCGCGNNNNNNNTTTTTTTTTTTTTTTTTTVN	C6_dual Biotin
97	PA_cell tag97_mol tag_(dT)18VN	CCATCTCATCCCTGCGTGTCTCCGACTCAGACACGCGNNNNNNNTTTTTTTTTTTTTTTTTTVN	C6_dual Biotin
98	PA_cell tag98_mol tag_(dT)18VN	CCATCTCATCCCTGCGTGTCTCCGACTCAGACGCGCGNNNNNNNTTTTTTTTTTTTTTTTTTVN	C6_dual Biotin
99	PA_cell tag99_mol tag_(dT)18VN	CCATCTCATCCCTGCGTGTCTCCGACTCAGCGTGTCGNNNNNNNTTTTTTTTTTTTTTTTTTVN	C6_dual Biotin
100	PA_cell tag100_mol tag_(dT)18VN	CCATCTCATCCCTGCGTGTCTCCGACTCAGCGTGACGNNNNNNNTTTTTTTTTTTTTTTTTTVN	C6_dual Biotin

※MGB stands for minor groove binder. It is a non-fluorescent quencher that increases the Tm of a probe because of its minor groove binding ability.

Supplementary Table 5 | Primer and probe sequences used in qPCR.

No.	Gene	Primer/Probe name	Seq. (5'→3')	Length (bp)	Tm (℃)	GC (%)
1	RPS18	RPS18 PCR FW	GTGTCCGAGGCCAGCACA	18	58.5	66.7
		RPS18 PCR RV	GACAAGGCCTACAGACTTATTTCTTC	26	58.7	42.3
		RPS18 MGB	CCGTGGGTGTGTCCAAG	17	57.4	64.7
2	GAPDH	GAPDH PCR FW	CACTGAATCTCCCCTCCTCACA	22	59.3	54.5
		GAPDH PCR RV	TACATGACAAGGTGCGGCTC	20	60.3	55
		GAPDH MGB	AGTTGCCATGTAGACCC	17	54.3	52.9
3	RPLP0	RPLP0 PCR FW	TCGGACGAGGATATGGGATT	20	59	50
		RPLP0 PCR RV	TGCAAATAAAACTGGCTAAGTTGG	24	58.1	37.5
		RPLP0 MGB	TCTCTTTGACTAATCACCAAAA	22	52.6	31.8
4	RPLP27	RPLP27 PCR FW	GGGAGGCCAAGGTCAAGT	18	59.2	61.1
		RPLP27 PCR RV	ACAAAGCATCTAAAACCGCAG	21	58.6	42.9
		RPLP27 MGB	AGAGATACAAGACAGGCAAG	20	55.9	45
5	RPLP2	RPLP2 PCR FW	ATGAGAAGAAGGAGGAGTCTG	21	57.6	47.6
		RPLP2 PCR RV	AATTTAATCAAAAAGGCCAAATCC	24	55.5	29.2
		RPLP2 MGB	AGAGTCAGATGATGACATGG	20	52.9	45
6	RPL4	RPL4 PCR FW	AAGAAGCCTGCTGCATAAAC	20	58	45
		RPL4 PCR RV	TCAAAAGAAGCTGTCCAAAATG	22	55.3	36.4
		RPL4 MGB	ATTATTCCATAAAGGTCAAA	20	48.7	25

7	RPLP1	RPLP1 PCR FW	AAGTGGAAGCAAAGAAAGAAGAATCC	26	59.9	38.5
		RPLP1 PCR RV	TGAACATGTTATAAAAGAGGTTTAGT	26	54.9	26.9
		RPLP1 MGB	AGGAGTCTGATGATGACATG	20	53	45
8	EEF1G	EEF1G PCR FW	AAAGCCTTCAATCAGGGCAA	20	57.4	45
		EEF1G PCR RV	GTTCAGTTTCCTTTAATGACCC	22	55	40.9
		EEF1G MGB	CATCTCTTGCCATCACCTA	19	52.4	47.4
9	RPS20	RPS20 PCR FW	GAGATTGTTAAGCAGATTACTTCCA	25	56.7	36
		RPS20 PCR RV	CTTAAGCATCTGCAATGGTG	20	54.4	45
		RPS20 MGB	TATTGAGCCAGGAGTTGAG	19	51.4	47.4
10	B2M	B2M PCR FW	ATTCATATTTACTTCTTATACATTTGA	27	51.9	18.5
		B2M PCR RV	AAGAGATAACACATCAAGTTT	21	51.9	28.6
		B2M MGB	GTTTTATGATTTATTTAACTTGTGG	25	59.6	24
11	RPL13A	RPL13A PCR FW	TCTAGAAGCAGAAATAGACTGGGAA	25	57.5	40
		RPL13A PCR RV	ATTTCTGATTACAAAATACAGGTGA	25	53.9	28
		RPL13A MGB	TTACAGGCATCGCCCATG	18	58.1	55.6
12	ATP5B	ATP5B PCR FW	CCCTAACCCAAAAAGCTTCATT	22	57.5	40.9
		ATP5B PCR RV	GAGGGGTGTACATTTTATTGGA	22	55.5	40.9
		ATP5B MGB	ACAAGAGCCTTGATTGA	17	50.7	41.2
13	ALDOA	ALDOA PCR FW	TTGCCCGCGCTCTTTCTTC	19	59.7	57.9
		ALDOA PCR RV	AAAGGGTGATGGACTTAGCATTCA	24	58.9	41.7
		ALDOA MGB	TGACAGTGGTGTGTGGTG	18	58	55.6
14	OAZ1	OAZ1 PCR FW	AGAAGTTTCTTATTTGGAGTCT	22	54.4	31.8
		OAZ1 PCR RV	GGTGTAATCACTTTATTGGCTT	22	55	36.4
		OAZ1 MGB	TTGGAAACAGTCTTAGTC	18	49.8	38.9
15	VIM	VIM PCR FW	AATCTTGTGCTAGAATACTT	20	51.2	30
		VIM PCR RV	AAGATTTATTGAAGCAGAACCA	22	54.4	31.8
		VIM MGB	AATACCATTAAAACTGCTT	19	49.5	26.3
16	TBP	TBP PCR FW	CTCCAGTATTGCAGGACAGA	20	57.7	50
		TBP PCR RV	AACTGCATTTATTGTACAGAGT	22	54.1	31.8
		TBP MGB	TAATGAAAATGAATGGCTGTAC	22	52.3	31.8
17	HPRT1	HPRT1 PCR FW	TAGTAGTGTTTCAGTAATGTTGACT	25	55.8	32
		HPRT1 PCR RV	TTAAAAGGGAACTGCTGACAAA	22	55	36.4
		HPRT1 MGB	ATTTTCCAACTTGTTCAAAT	20	50.9	25
18	SDHA	SDHA PCR FW	CCAGGGAGCGTGGCACTT	18	62.2	66.7
		SDHA PCR RV	AAGAGCTGTGCCCAGTTTTATC	22	59	45.5
		SDHA MGB	CCTTTGTCCCTTGCTTCA	18	54.8	50
19	GUSB	GUSB PCR FW	CGTTTCTGGCCTGGGTTTTG	20	59.6	55
		GUSB PCR RV	TCAGTAGCCACTTTCATGCC	20	57.7	50
		GUSB MGB	TCTATTCTAGCAGGGAACAC	20	52.7	45
20	HMBS	HMBS PCR FW	GATGACTGCCTTGCCTCCTC	20	60.3	60
		HMBS PCR RV	CAGAACTGGTTTATTAGTAGGATTGG	26	58.1	38.5
		HMBS MGB	AAGCAACAGCCTTTGAATG	19	55.5	42.1

Supplementary Table 6 | PCR primer sequences for ϕX174.

No.	Name	seq.(5'→3')	Length (bp)	Tm (℃)	GC(%)
1	X174_Cont. FW_3483_T7Pro.	TAATACGACTCACTATAGGGCGCCAGAATACGAAAGACC	39	62.8	52.6
2	X174_Cont. RV_3805_Oligo(dT)30	TTTTTTTTTTTTTTTTTTTTTTTTTTTTTTGAAGCAGCATCAGTGACGAC	50	63.1	55

Supplementary Table 7 | The qPCR primer sequences and MGB probes for ϕX174.

No.	Name	seq.(5'→3')	Length (bp)	Tm (℃)	GC(%)
1	X174_PCR. FW_3642	ACGGCTGGTCAGTATTTTAC	20	59.3	45
2	X174_PCR. RV_3728	CGTTTGCTGATGAACTAAGTC	21	60	42.9
3	X174_MGB Probe_3684	ACTCGCAAGGTTAGTGCT	18	57.1	50

Supplementary Table 8 | Primer sequences for standard DNA preparations.

No.	Gene	Primer name	Seq. (5'→3')	Length (bp)	Tm (℃)	GC (%)
1	ATP5B	Con_ATP5B FW	TGCACGGAAAATACAGCGTTT	21	59.9	42.9
2		Biotin ATP5B RV	GAGGGGTGTACATTTTATTGGA	22	55.5	40.9
3	GAPDH	Con_GAPDH FW	CAGCCCCAGCAAGAGCACAA	20	61.7	60
4		Biotin GAPDH RV	TACATGACAAGGTGCGGCTC	20	60.3	55
5	GUSB	Con_GUSB FW	CTGATACCACCTGCGTGTCC	20	59.5	60
6		Biotin GUSB RV	TCAGTAGCCACTTTCATGCC	20	57.7	50
7	HMBS	Con_HMBS FW	CTGTCCAGTGCCTACATCCC	20	59.3	60
8		Biotin HMBS RV	CAGAACTGGTTTATTAGTAGGATTGG	26	58.1	38.5
9	HPRT1	Con_HPRT1 FW	ACTGTTTATTTGCACTATGAGCC	23	58	39.1
10		Biotin HPRT1 RV	TTAAAAGGGAACTGCTGACAAA	22	55	36.4
11	RPL4	Con_RPL4 FW	GCCAGGAATCACAAGCTCCG	20	58.5	60
12		Biotin RPL4 RV	TCAAAAGAAGCTGTCCAAAATG	22	55.3	36.4
13	RPLP1	Con_RPLP1 FW	CCTCATTAAAGCAGCCGGTGT	21	60	52.4
14		Biotin RPLP1 RV	TGAACATGTTATAAAAGAGGTTTAGT	26	54.9	26.9
15	RPS18	Con_RPS18 FW	TATGCAGAATCCACGCCAGT	20	59	50
16		Biotin RPS18 RV	GACAAGGCCTACAGACTTATTTCTTC	26	58.7	42.3
17	RPL13A	Con_RPL13A FW	TCTTAGTCACTGCCTCCCGAA	21	59.5	52.4
18		Biotin RPL13A RV	ATTTCTGATTACAAAATACAGGTGA	25	53.9	28
19	RPS20	Con_RPS20 FW	CCTAACAAGCCGCAACGTA	19	57.4	52.6
20		Biotin RPS20 RV	CTTAAGCATCTGCAATGGTG	20	54.4	45
21	ALDOA	Con_ALDOA FW	CTTCGTCTCTAACCACGCCTA	21	60.3	52.4
22		Biotin ALDOA RV	AAAGGGTGATGGACTTAGCATTCA	24	58.9	41.7
23	B2M	Con_B2M FW	AACTCTTCAATCTCTTGCACT	21	57	38.1
24		Biotin B2M RV	AAGAGATAACACATCAAGTTT	21	51.9	28.6

No.	Gene	Primer name	Seq. (5'→3')	Length (bp)	Tm (℃)	GC (%)
25	EEF1G	Con_EEF1G FW	CTTCGCCAGTGTCATCCTT	19	58.2	52.6
26		Biotin EEF1G RV	GTTCAGTTTCCTTTAATGACCC	22	55	40.9
27	SDHA	Con_SDHA FW	CTTCATACGCTTCTGCACTCT	21	59.7	47.6
28		Biotin SDHA RV	AAGAGCTGTGCCCAGTTTTATC	22	59	45.5
29	TBP	Con_TBP FW	TGATGCCCTTCTGTAAGTGCC	21	60.3	52.4
30		Biotin TBP RV	AACTGCATTTATTGTACAGAGT	22	54.1	31.8
31	VIM	ConVIM FW	AGAAACAGCTTTCAAGTGCCT	21	60.4	42.9
32		Biotin VIM RV	AAGATTTATTGAAGCAGAACCA	22	54.4	31.8
33	RPLP0	Con_RPLP0 FW	GGAAACTCTGCATTCTCGCTTC	22	58.9	50
34		Biotin RPLP0 RV	TGCAAATAAAACTGGCTAAGTTGG	24	58.1	37.5
35	RPLP2	Con_RPLP2 FW	CTACGTCGCCTCCTACCTG	19	59.1	63.2
36		Biotin RPLP2 RV	AATTTAATCAAAAAGGCCAAATCC	24	55.5	29.2
37	RPLP27	Con_RPLP27 FW	CTAATGCCCACAAGGTACTCT	21	57.6	47.6
38		Biotin RPLP27 RV	ACAAAGCATCTAAAACCGCAG	21	58.6	42.9
39	OAZ1	Con_OAZ1 FW	TTGTCCGCATGTTGTAATCGT	21	58.9	42.9
40		Biotin OAZ1 RV	GGTGTAATCACTTTATTGGCTT	22	55	36.4

Supplementary Table 9| Results of bead-seq for 21 markers for PMA induction.

Descending order	Ensembl gene ID	Gene name	Slope	SD	Marker index
1	ENSG00000095303	PTGS1	4.844	5.664094533	0.855148634
2	ENSG00000148180	GSN	11.62	29.59488015	0.392631396
3	ENSG00000171476	HOPX	0.588	1.623686606	0.361833256
4	ENSG00000136689	IL1RN	21.755	67.01541284	0.324619935
5	ENSG00000197262	CCL4L2	1.695	5.77770389	0.293425965
6	ENSG00000160255	ITGB2	14.776	51.65735971	0.286036963
7	ENSG00000157483	MYO1E	1.162	4.146117261	0.2802171
8	ENSG00000184271	POU6F1	0.29	1.054738888	0.274577576
9	ENSG00000156535	CD109	0.338	1.3552308	0.249371305
10	ENSG00000143119	CD53	6.443	25.83916258	0.24933297
11	ENSG00000249992	TMEM158	0.335	1.363649236	0.245740253
12	ENSG00000163565	IFI16	0.603	2.526715369	0.238470926
13	ENSG00000161570	CCL5	9.519	39.97309538	0.238139632
14	ENSG00000134531	EMP1	6.093	25.98633521	0.234454505
15	ENSG00000128965	CHAC1	0.24	1.098409224	0.218068515
16	ENSG00000142669	SH3BGRL3	20.182	95.74201948	0.210797272
17	ENSG00000121769	FABP3	0.768	3.693263693	0.207853757
18	ENSG00000118985	ELL2	1.186	5.727007573	0.207124332
19	ENSG00000157227	MMP14	0.835	4.040131343	0.2065873
20	ENSG00000197461	PDGFA	0.695	3.507213234	0.198057796
21	ENSG00000118785	SPP1	258.895	1770.996532	0.146186059

Supplementary Table 10 | Types of reads and their definitions.

Types of reads	Description in the code	Detailed Description
No cell tag	no_cell_tag	No matching cell-ID tag at predetermined position.
No mol tag	no_mol_tag	The acquired sequence is shorter than the set length of the cell-ID tag and UMI tag in a configure file.
No poly-T	no_poly_t	No poly-T, defined by normal expression as /^([ACGT]{0,2TTTTTT+)([ACGTN]*)$/.
Poly-T only	poly_t_only	No mRNA sequence following poly-T.
Too short	too short	The acquired sequence of mRNA following poly-T is shorter than the set value in the configure file.
Target genes	authentic_target	The acquired sequence of mRNA was aligned uniquely on the target genes by BLAST.
Other genes	authentic_secondary	The acquired sequence was aligned on a part of all mRNA sequences (Ensembl v.79) by Bowtie 2.
Others	authentic_mystery	The acquired sequence was not categorized to any type of reads.

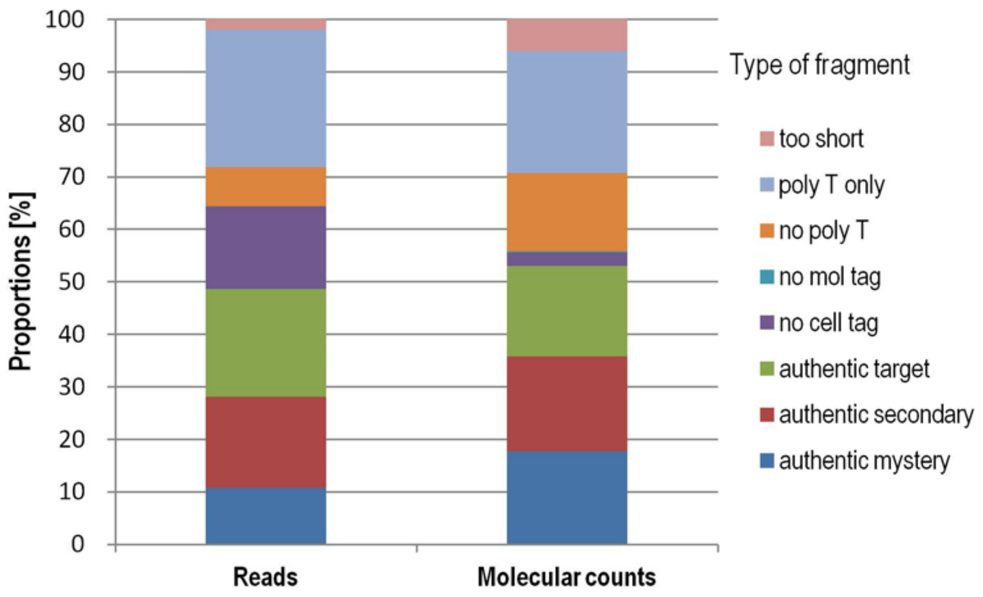

Supplementary Figure 10 | Proportions of types of reads and derived molecular counts.

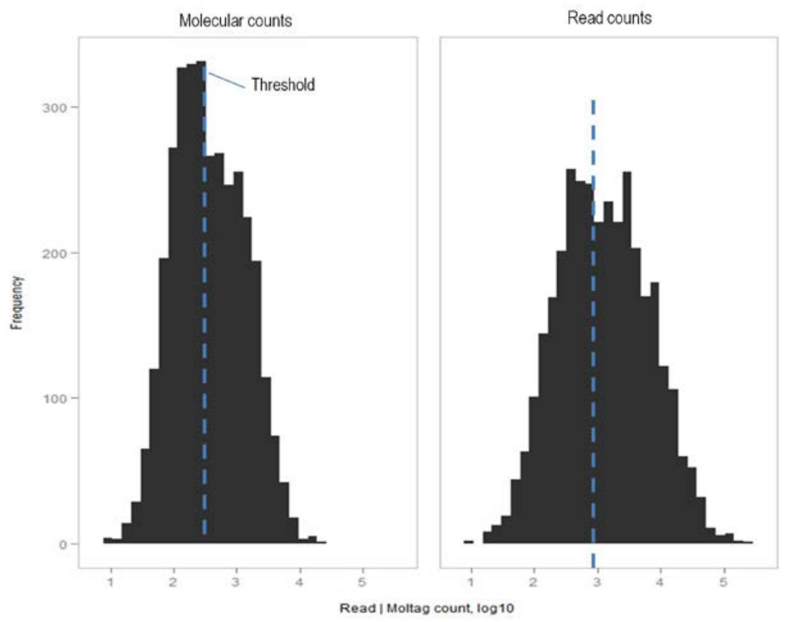

Supplementary Figure 11 | Histograms of molecular counts and read counts. The thresholds indicate the microchambers that contain cells.

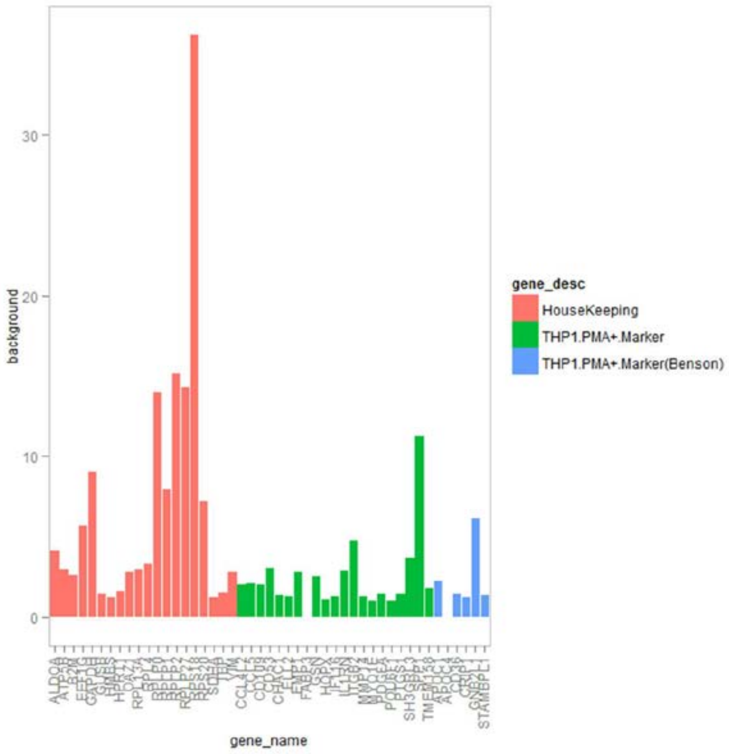

Supplementary Figure 12 | Background profiles for subtraction, as determined by averaging the microchambers below the threshold.

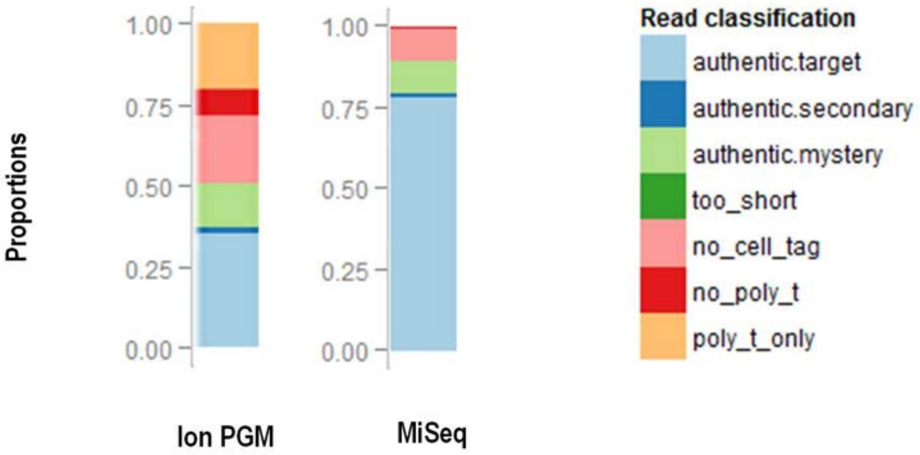

Supplementary Figure 13 | Comparison of the proportions of the types of reads for two types of sequencing platforms.

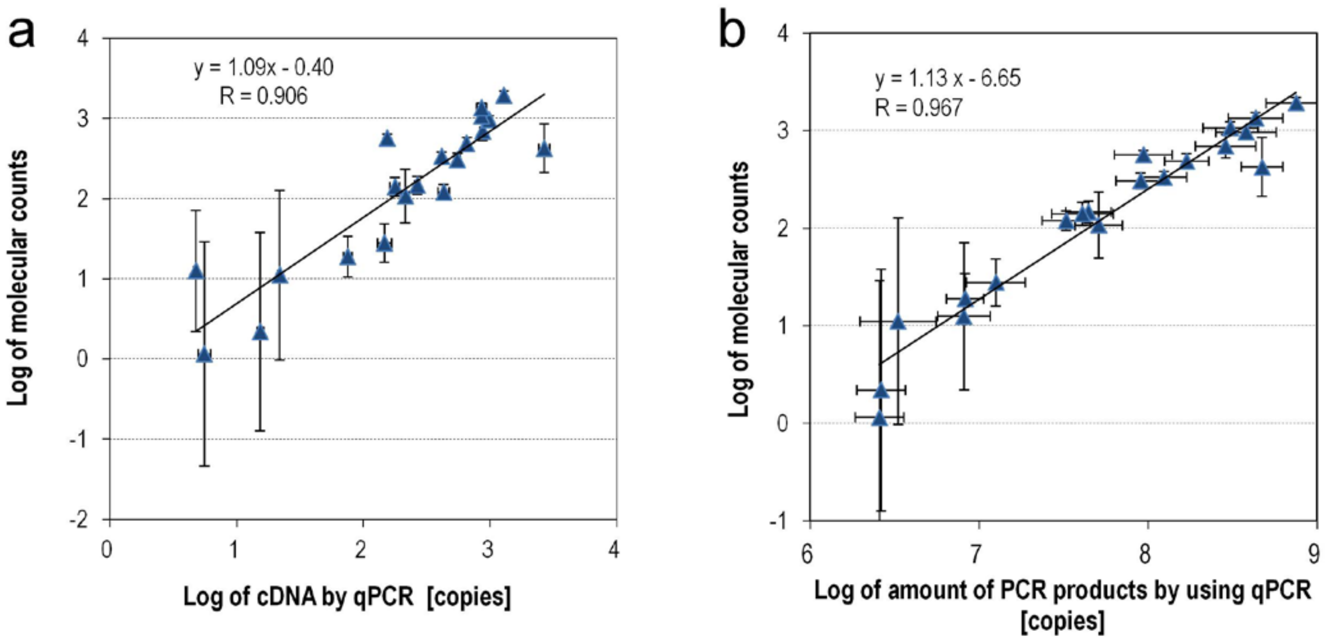

Supplementary Figure 14 | Correlations between molecular counts and cDNAs or PCR products for housekeeping genes in pooled mRNA samples.

Supplementary Table 11 | Direct evaluation of RT efficiency from cRNA on VFACs. The model cRNA of ϕX174 was injected into the microchambers, and RT-qPCR quantification was performed using a previously described method.

Amount of injected cRNA in a chamber [copies]	Number of chambers on a VFAC [copies]	Amount of injected cRNA on a VFAC [copies]	Amount of synthesized cDNA on a VFAC [copies](RT-qPCR)	Standard error [copies]	Efficiency of cDNA synthesis [%] (one-sided 84% confidence interval)
2.0E+04	50	1.0E+05	1.13E+05	2.3E+04	90

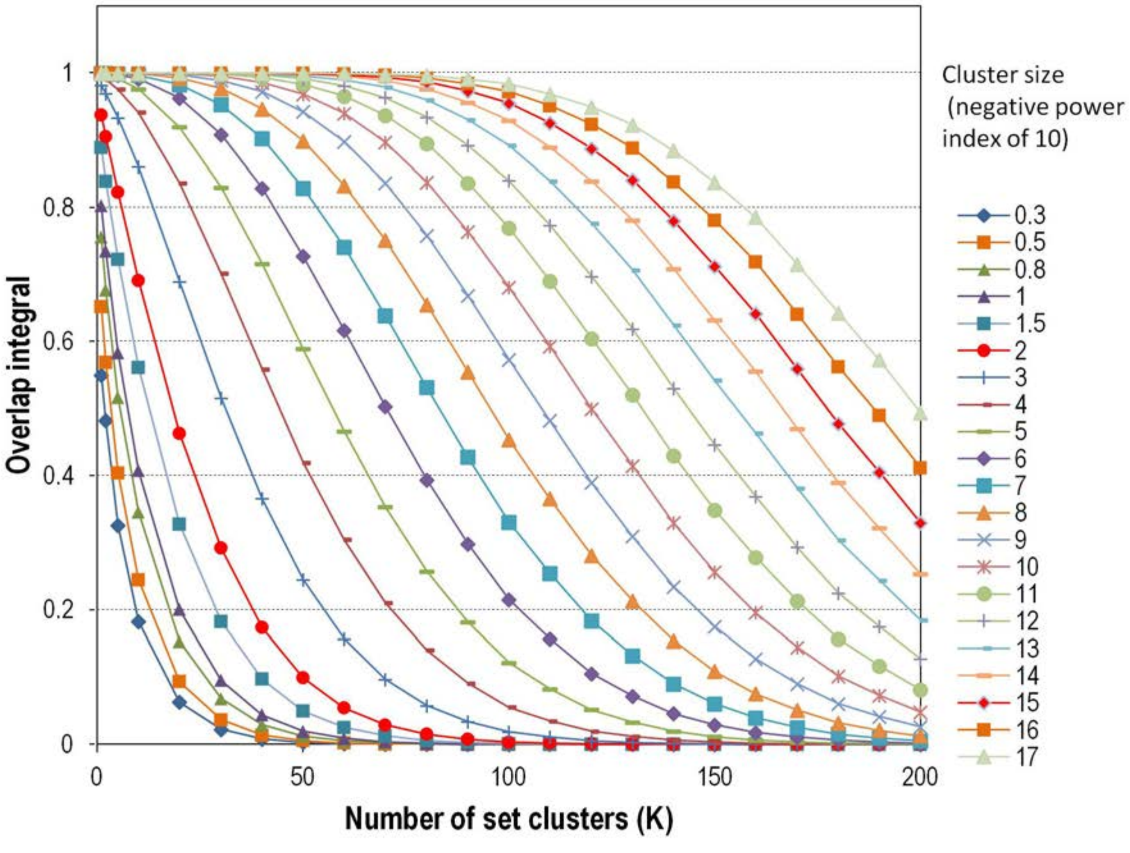

Supplementary Figure 15 | Overlap integrals for various dimensions and cluster sizes (CS). The overlap integrals were calculated as follows. First, data points were generated following a normal distribution at the origin in n dimensions. Second, for each generated point (x_i), virtual data were generated following a normal distribution at x_i. Finally, the Mahalanobis distances between the generated virtual data and the origin were calculated, and the probability of virtual data inside CS was taken as the overlap integral.

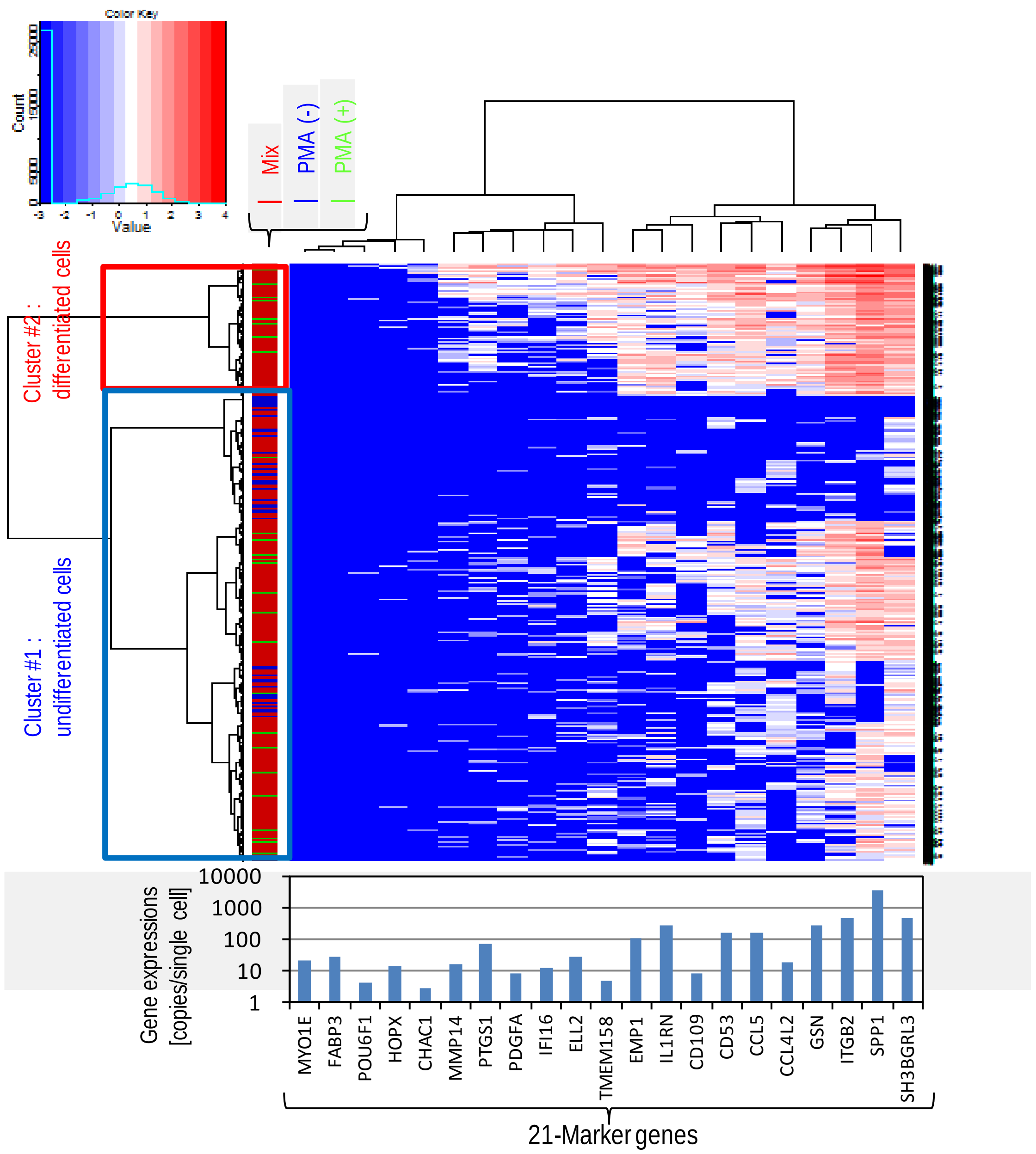

Supplementary Figure 16 | Heat map of 21 marker expression levels in 1967 cells. Two or three types of undifferentiated cells are evident, as supported by the pq-values for 10 marker genes (Supplementary Fig. 8). Bar chart shows average gene expression levels evaluated by RNA-seq at the single-cell level (Bead seq [36])

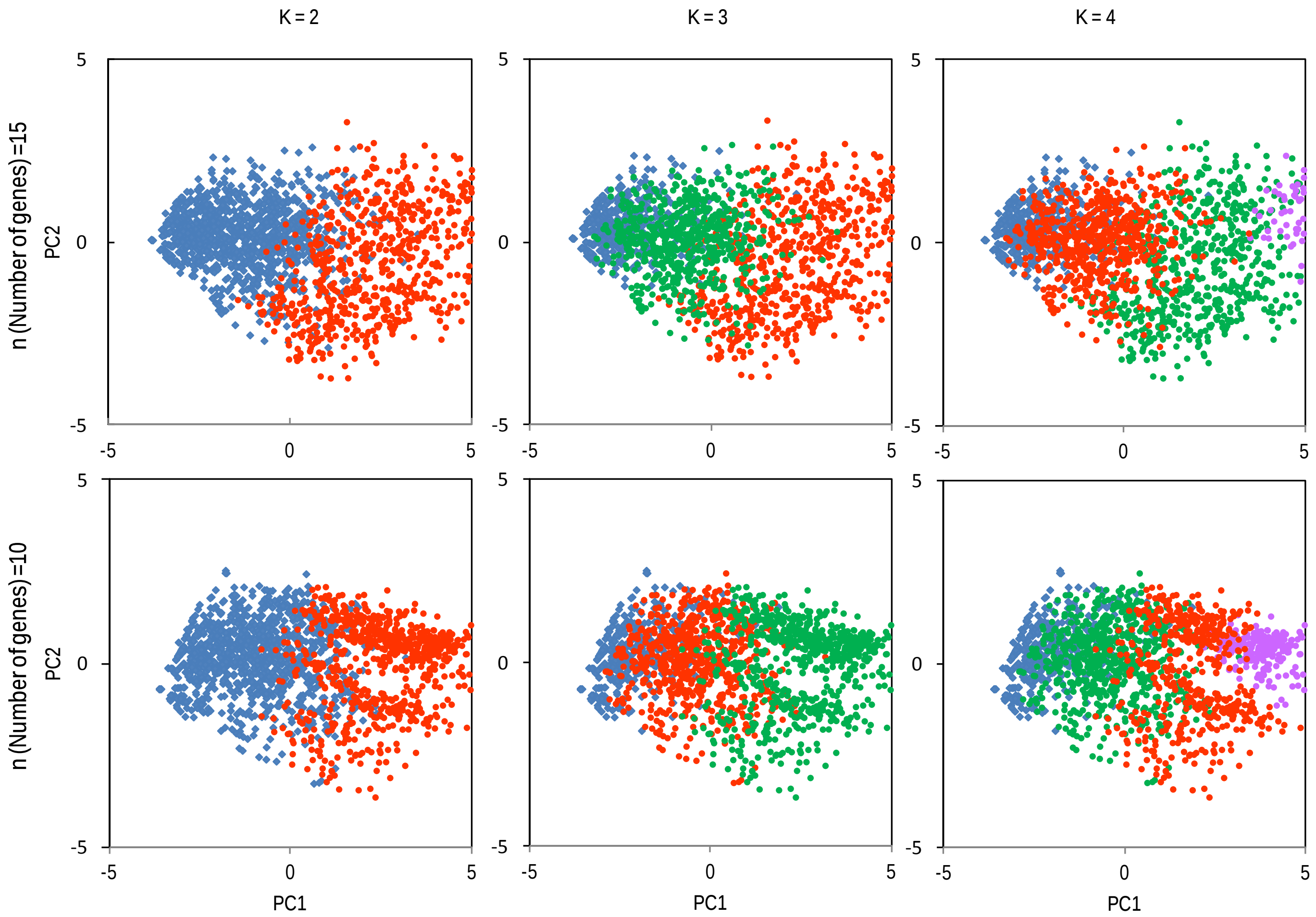

Supplementary Figure 17 | Visualization of clusters for 10 and 15 marker genes based on PCA.
